# The root‐knot nematode effector Mi1D08B targets a NINJA‐family co‐repressor to suppress jasmonate production and promote infection in soybean

**DOI:** 10.1111/tpj.71095

**Published:** 2026-08-22

**Authors:** Peitong Li, Sobhan Bahrami Zadegan, Nicole Coffey, Nandiny Ghosh, J. Hollis Rice, Ahmad El‐Messidi, Mohamed Boshnag, Tabibul Islam, Tessa Burch‐Smith, Vince Pantalone, Tarek Hewezi

**Affiliations:** ^1^ Department of Plant Sciences University of Tennessee Knoxville Tennessee 37996 USA; ^2^ UT‐ORNL Graduate School of Genome Science and Technology, University of Tennessee Knoxville Tennessee 37996 USA; ^3^ Donald Danforth Plant Science Center St Louis Missouri 63132 USA

**Keywords:** soybean, root‐knot nematode, *Meloidogyne incognita*, Mi1D08B effector, jasmonic acid, OPDA, NINJA co‐repressor

## Abstract

Plant‐parasitic nematodes deploy secreted effector proteins that reprogram host cellular processes to establish parasitism. Here, we characterized Mi1D08B, a putative dorsal‐gland effector from the root‐knot nematode *Meloidogyne incognita* and defined its role in subverting soybean jasmonate‐mediated immunity. Mi1D08B is conserved across several *Meloidogyne* species and localizes to the plant nucleus and cytoplasm. Overexpression of *Mi1D08B* significantly increased galling and egg production, demonstrating a strong virulence role. A gall‐specific yeast two‐hybrid screen identified the NINJA‐family co‐repressor mc410 as a host target of Mi1D08B. Consistent with the functional relevance of this interaction, *mc410* promoter activity was detected in galls and giant cells throughout nematode infection. Genetic manipulation of *mc410* phenocopied Mi1D08B activity as *mc410* overexpression enhanced susceptibility, whereas *mc410* silencing reduced nematode infection. Hormone profiling revealed that *Mi1D08B* overexpression elevated 12‐oxo‐phytodienoic acid (OPDA) but reduced jasmonic acid (JA) and JA‐Ile, consistent with a bottleneck at the peroxisomal OPDA‐to‐JA conversion. Furthermore, several metabolites associated with this conversion were reduced, indicating that Mi1D08B perturbs metabolic flux through the peroxisomal phase of jasmonate biosynthesis. Gene expression analyses supported this biochemical signature, with upregulation of plastidial OPDA‐biosynthetic genes and downregulation of peroxisomal OPDA‐reductases, JA‐conjugating enzymes, and JA‐responsive markers. Together, our data support a model in which Mi1D08B effector interacts with a NINJA co‐repressor to suppress JA biosynthesis and signaling, thereby coupling nuclear transcriptional repression to altered metabolism. This mechanism deepens our understanding of nematode manipulation of host hormone networks and highlights the Mi1D08B–mc410 interface and OPDA conversion as promising targets for engineering nematode resistance.

## Significance Statement

Plant‐parasitic nematodes rely on secreted effectors to suppress host defenses, but how these molecules disrupt hormone‐mediated defense pathways is poorly understood. We show that the *Meloidogyne incognita* effector Mi1D08B targets the NINJA‐family co‐repressor mc410 to suppress jasmonate biosynthesis and signaling, revealing a key host–pathogen interface that could be exploited for nematode resistance.

## INTRODUCTION

Plant‐parasitic nematodes (PPNs) rank among the most destructive plant pathogens worldwide, posing a major global threat to agricultural productivity and food security. Root‐knot nematodes (RKNs; *Meloidogyne* spp.), including *M. incognita*, *M. arenaria*, *M. hapla*, *M. javanica*, and *M. enterolobii*, are sedentary endoparasites that penetrate host roots and establish long‐lived feeding sites (Escobar et al., 2015; Moens et al., 2009; Sharma & Chaubey, 2023). RKNs have an exceptionally broad host range and infest thousands of vascular plant species ranging from major field crops such as soybean, potato, tomato, and cotton to numerous horticultural and ornamental plants, causing significant reductions in both yield and quality (Jones et al., 2013). Among RKN species, *M. incognita* is one of the most widespread and economically important, and its impact on soybean production is particularly severe. Losses from RKNs in soybean are second only to those caused by the soybean cyst nematode (*Heterodera glycines*). In the United States, recent estimates for 2020–2024 attribute an average annual loss of 12.6 million bushels (≈USD 154 million) in soybean production to RKNs (Crop Protection Network, 2024). Rising temperatures associated with environmental changes are expected to intensify RKN pressure by accelerating their life cycle and increasing damage to soybean production (Ghini et al., 2008; Kantor et al., 2025). Notably, there are currently no commercially available soybean cultivars with strong resistance to RKN, underscoring the limitations of existing management strategies. Therefore, a detailed understanding of the molecular mechanisms that enable RKNs to parasitize their hosts is essential for the development of sustainable and durable control approaches.

RKNs complete their life cycle in approximately 20–40 days, depending on temperature, soil moisture, host species, and other environmental factors. The cycle begins with the hatching of second‐stage juveniles (J2s) from eggs. These infective juveniles are attracted to host roots by CO_2_, acidic compounds, and carbohydrates in root exudates (Berliner et al., 2023; Bird, 1959; Hewezi & Baum, 2017; Tsai et al., 2021). After locating a suitable host, J2s penetrate the root elongation zone and migrate intercellularly toward the vascular cylinder, where they establish permanent feeding sites known as giant cells. Following giant cell establishment, nematodes undergo three successive molts to the third‐ and fourth‐stage juveniles (J3 and J4) and ultimately develop into adult females or males. Females produce gelatinous egg masses containing numerous eggs with first‐stage juveniles (J1), which molt to J2s within the egg and hatch to initiate the next generation (Escobar et al., 2015).

Although recent studies have revealed that a small subset of RKN effectors can function as avirulence factors recognized by host proteins (Gleason et al., 2008; Lozano‐Torres et al., 2012; Rutter et al., 2014; Zadegan et al., 2026), the majority of characterized nematode effectors act as virulence determinants that manipulate host immunity, development, and metabolism to promote susceptibility and successful parasitism (Bali & Gleason, 2024; Hewezi & Baum, 2013; Mejias et al., 2019; Rutter et al., 2022). These effectors perform a wide, complementary suite of functions that enable invasion, feeding‐site formation, suppression or reprogramming of host defenses and development, and manipulation of host metabolism. For example, classical secreted cell‐wall‐degrading enzymes, such as cellulases and pectate lyases, are major components of the RKN secretome that soften and degrade plant cell walls to facilitate root penetration and intracellular migration (Hewezi, 2015; Wieczorek, 2015). Early secreted effectors play a critical role in establishing infection by dampening host immunity. For example, MiMsp40 suppresses PTI/ETI signaling during the early stages of parasitism (Niu et al., 2016), while the secreted Mi‐CRT, MiEFF12, and MiCE108 broadly attenuate plant immune responses to promote nematode success (Jaouannet et al., 2013; Soulé et al., 2024; Yu et al., 2024). Secreted peptide effectors manipulate host developmental pathways to enhance susceptibility. For instance, 16D10 stimulates root growth by modulating host transcription factor activity (Huang et al., 2006), while peptide mimics such as MiIDL1 and CLE‐like effectors imitate endogenous plant signals to hijack developmental signaling processes (Hu & Hewezi, 2018; Kim et al., 2018; Rutter et al., 2014). Reprogramming host transcription and alternative splicing events have been also reported as fundamental functions of RKN effector as in the case of MiEFF18, MiMSP8, and Mi2G02 effectors (Chen et al., 2025; Hewezi, 2024; Mejias et al., 2021; Zhao et al., 2024).

Recent studies show that RKN effectors target jasmonate (JA) signaling at multiple levels, including biosynthesis, hormone perception, transcriptional repression, and JA–SA cross talk, thereby compromising a key pathway of plant defense against RKNs. JA is synthesized through the octadecanoid pathway beginning with linoleic acid (18:2) and α‐linolenic acid (18:3) from the chloroplast membrane by phospholipases (Browse, 2009; León & Sánchez‐Serrano, 1999). The first step is catalyzed by allene oxide synthase (AOS) and allene oxide cyclase (AOC) to generate 12‐oxo‐phytodienoic acid (OPDA), a key precursor that accumulates in response to biotic stress (Chini et al., 2016; Han, 2017). OPDA is subsequently translocated into the peroxisome, where it is reduced to JA by OPDA reductase (OPR) (Browse, 2009; Nilsson et al., 2016; Yi et al., 2024). While OPR3 has been considered the main enzyme responsible for converting OPDA to JA, recent studies have shown that OPR2 can catalyze an alternative JA‐biosynthetic pathway, which converts the OPDA‐derived intermediate 4,5‐ddh‐JA to JA (Chini et al., 2018; Verhoeven et al., 2023). These studies highlight that OPR2 and OPR3 act in parallel and make independent contributions to JA production. JA is further conjugated to isoleucine to form JA‐Ile (the active form) and perceived by the SCF/COI1 receptor complex, triggering degradation of JAZ repressors and release of transcription factors (e.g., MYC2) that activate canonical JA‐dependent defense genes (Johnson et al., 2023; Kim et al., 2025; Roychowdhury et al., 2025). Because JA signaling must be tightly balanced to defend against pathogens while minimizing fitness costs to the host, plants rely on dedicated repression machinery to fine‐tune JA responses. Repression of these JA responses is mediated by the JAZ–NINJA–TPL module where NINJA (Novel Interactor of JAZ) proteins are central components. Under non‐inducing conditions NINJA links JAZ repressors to the TOPLESS/TPL‐related co‐repressors via its EAR repression motif, stabilizing a MYC–JAZ–NINJA–TPL complex that keeps JA‐responsive genes off (Pauwels et al., 2010; Zhou et al., 2023).

Nematode effectors exploit both biosynthetic and repression nodes to silence JA defenses. For example, *M. incognita* MiMSP32 binds host OPR2 and elevates OPDA levels while blocking its reduction toward JA, implicating direct suppression of JA biosynthesis (Verhoeven et al., 2023). Other effectors interfere with JA perception and signaling. The RKN nuclear effector MiISE23 stabilizes JAZ repressors to prevent JA‐Ile–dependent activation of defense genes (Shi et al., 2025). A similar JAZ‐stabilizing activity was suggested for the *M. javanica* Mj2G02 effector, leaving JA‐responsive defenses suppressed (Song et al., 2021). In addition, host targets of the MiEFF1 effector have been shown to regulate the expression of SA and JA defense‐related genes despite the underlying mechanism remaining unknown (Truong et al., 2021).

Collectively, these studies establish JA signaling as a central defense hub whose timely activation is critical for RKN resistance, and disruption of JA biosynthesis, perception, or repression produces large effects on plant susceptibility. In this study, we demonstrate that the *M*. *incognita* effector Mi1D08B directly targets a soybean NINJA‐family co‐repressor, and this interaction is associated with OPDA accumulation and a net reduction in JA levels, consistent with an impairment of peroxisomal OPDA to JA conversion. Thus, Mi1D08B imposes a dual assault on host defenses by co‐opting the JAZ–NINJA repression machinery while also diminishing jasmonate biosynthesis, together suppressing JA‐dependent defense transcription to promote parasitism. By connecting a specific effector to a core JA repression component and demonstrating its impact on plant susceptibility and hormone metabolism, our work reveals a previously unrecognized mechanism by which RKNs simultaneously manipulate JA biosynthesis and signaling to suppress host defenses.

## RESULTS

### In silico analysis

**Figure 1:**
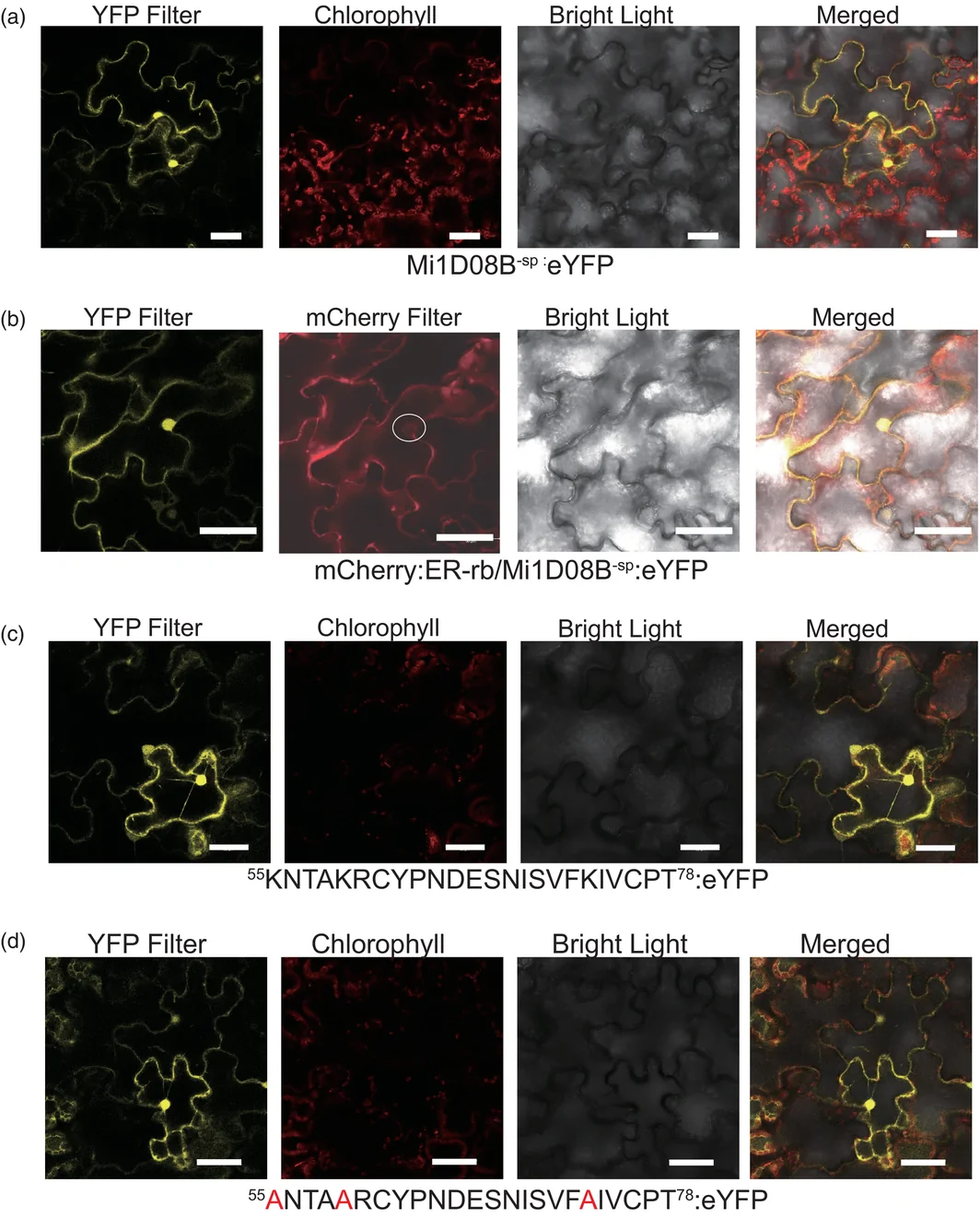
Subcellular localization of Mi1D08B constructs in *Nicotiana benthamiana* cells. (a) Representative confocal image of Mi1D08B^‐sp^:eYFP (mature protein lacking the signal peptide) showing fluorescence concentrated in the nucleus and the cytoplasm. (b) Co‐expression of Mi1D08B^‐sp^:eYFP with the ER marker ER‐rb (CD3‐960) shows fluorescence in the perinuclear ER (circled), supporting the nuclear localization of Mi1D08B. (c) YFP fused to the predicted bipartite NLS (⁵⁵KNTAKRCYPNDESNISVFKIVCPT⁷⁸) localizes to both the nucleus and the cytoplasm, mirroring the full‐length Mi1D08B^‐sp^:eYFP pattern. (d) YFP fused to the mutated NLS variant (⁵⁵ANTAARCYPNDESNISVFAIVCPT⁷⁸) also displays nuclear and cytoplasmic localization, indicating the predicted NLS is not solely responsible for nuclear accumulation. Images are representative of 3 independent replicates. Fluorescence channels are indicated on the images. Scale bars = 50 μm.

*Mi1D08B* (GenBank accession number AAR37368.1) is an uncharacterized dorsal‐gland putative effector encoding a 171 amino acid protein with an N‐terminal signal peptide (amino acids 1–18) for secretion (Huang et al., 2004). Sequence analysis revealed the presence of two canonical N‐glycosylation motifs (NKT^49‐51^ and NIS^68‐70^), suggesting that this nematode effector undergoes ER‐mediated glycosylation, which may contribute to its stability, secretion, and ability to function within the host plant cells during parasitism. Homology searches using BLAST identified homologous proteins in *Meloidogyne incognita*, *M. javanica*, *M. arenaria*, and *M. enterolobii* indicating that *Mi1D08B* belongs to a highly conserved effector family within closely related RKN species. More distantly related species, including *M. graminicola* and *M. hapla*, showed only 30–40% sequence identity. No sequence similarity to cyst nematodes was detected, suggesting that *Mi1D08B* represents an RKN‐specific secreted effector family. Interestingly, a moderate‐scoring bipartite nuclear localization signals (NLS) motif (^55^KNTAKRCYPNDESNISVFKIVCPT^78^) was predicted within the *Mi1D08B* protein, which may drive the secreted protein into the host nucleus. To investigate its subcellular distribution, the mature Mi1D08B protein without the N‐terminal signal peptide (Mi1D08B^‐sp^) was fused to enhanced yellow fluorescent protein (eYFP) and transiently expressed in *Nicotiana benthamiana* leaves. Confocal imaging showed that Mi1D08B^‐sp^:eYFP localized predominantly to the nucleus and the cytoplasm (Figure 1a). Co‐expression of Mi1D08B^‐sp^:eYFP with the ER‐resident marker ER‐rb (CD3‐960) (Nelson et al., 2007) showed that the perinuclear fluorescence corresponds to the ER marker surrounding the nucleus, further supporting the nuclear localization of Mi1D08B (Figure 1b). To determine whether the predicted NLS is responsible for nuclear targeting, the NLS sequence and a mutated variant (^55^ ANTAARCYPNDESNISVFAIVCPT^78^) were individually fused to YFP and transiently expressed in *N. benthamiana* leaves. Confocal analysis showed that both constructs displayed nuclear and cytoplasmic localization patterns comparable to that observed for the full‐length Mi1D08B^‐sp^:eYFP fusion (Figure 1c,d). These findings indicate that the predicted NLS motif is not responsible for the nuclear accumulation of Mi1D08B. Instead, its nuclear localization may occur through passive diffusion through nuclear pores, which is possible for relatively small proteins such as Mi1D08B.

### Overexpression of *Mi1D08B* ^*‐sp*^ effector increased soybean susceptibility to *M. incognita*

**Figure 2:**
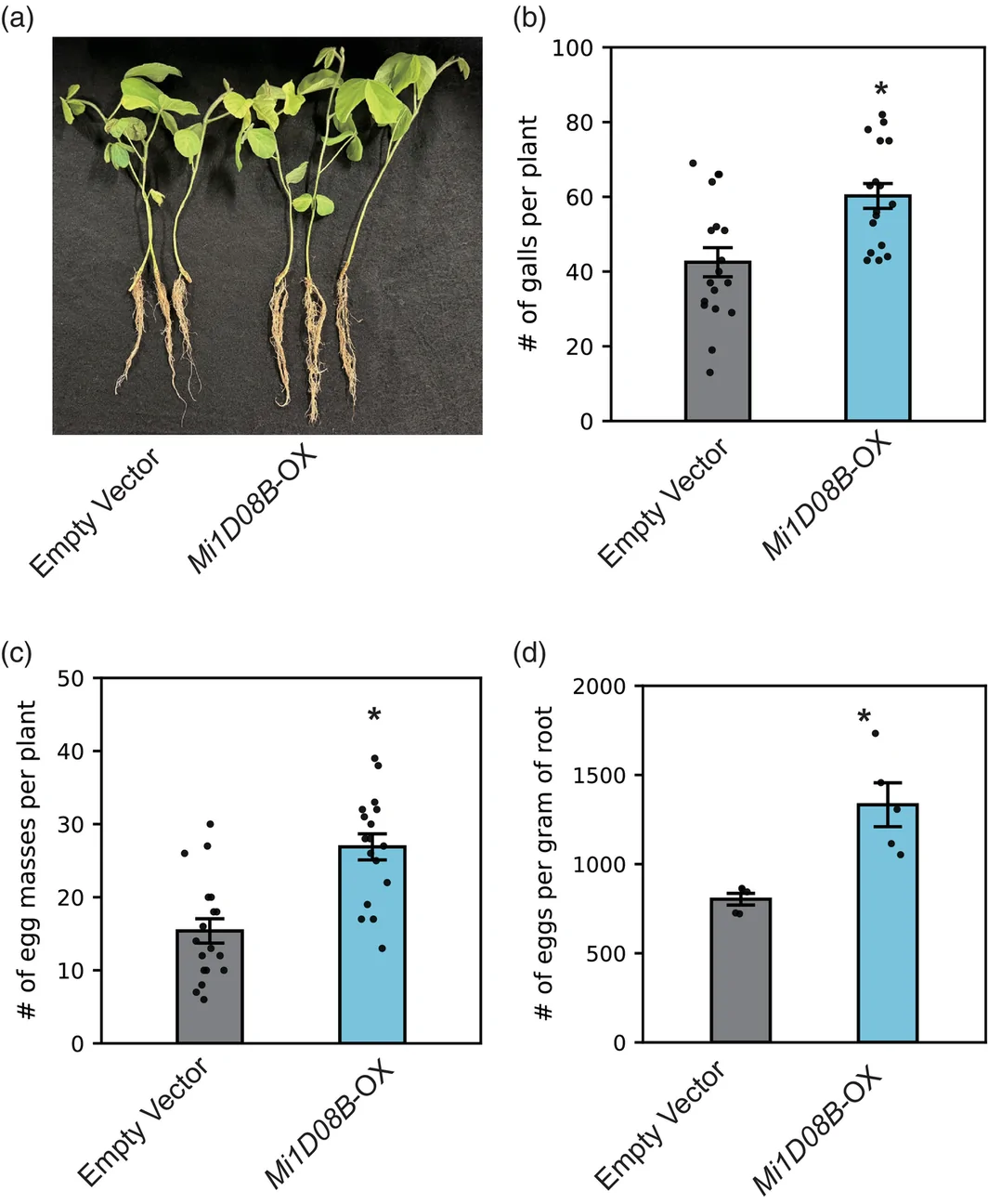
*Mi1D08B* overexpression increases soybean susceptibility to *Meloidogyne incognita*. (a) Representative root phenotypes of composite plants overexpressing Mi1D08B (*Mi1D08B*‐OX) compared with control plants expressing the empty vector. (b–d) Number of galls per plant (b), egg masses per plant (c), and eggs per gram of root tissues (d) at 5 weeks after inoculation with *M. incognita* juveniles. Dots represent individual biological replicates; bars show mean ± SE. Asterisks indicate statistically significant differences between *Mi1D08B*‐OX and empty vector control plants (*P* < 0.05, *t*‐test). Sample sizes: (b, c), empty vector (*n* = 18) and *Mi1D08B*‐OX (*n* = 17); (d) *n* = 5 per group. Raw data for gall numbers, egg mass numbers, and eggs per gram of root tissues in *Mi1D08B*‐overexpressing and control plants are provided in Table S1.

To determine the potential role of *Mi1D08B* in nematode pathogenicity, we overexpressed *Mi1D08B* ^*‐sp*^ coding sequences under the control of the soybean ubiquitin promoter in the susceptible soybean cultivar Williams 82 using a transgenic hairy root system. Composite plants with transgenic hairy roots were evaluated for any developmental defects. No apparent phenotypic differences were observed between the *Mi1D08B* ^*‐sp*^‐overexpressing plants and the control plant expressing the empty binary vector (Figure 2a). The expression level of *Mi1D08B* was quantified in the transgenic roots using RT‐qPCR analysis. *Mi1D08B* showed a high expression level comparable to the highly expressed reference gene *Actin*, confirming the function of the overexpression construct (Figure S1). Interestingly, overexpression of the *Mi1D08B* ^*‐sp*^ significantly increased soybean susceptibility to *M. incognita* compared with empty vector control lines (Figure 2b–d). The average number of galls increased by 42%, egg masses by 75%, and eggs per gram by 66% across approximately 20 replicate plants (Figure 2b–d). These results indicate that the *Mi1D08B* effector plays a vital role in facilitating successful nematode parasitism.

### Mi1D08B ^‐sp^ interacts with NINJA‐family mc410 in yeast

**Figure 3:**
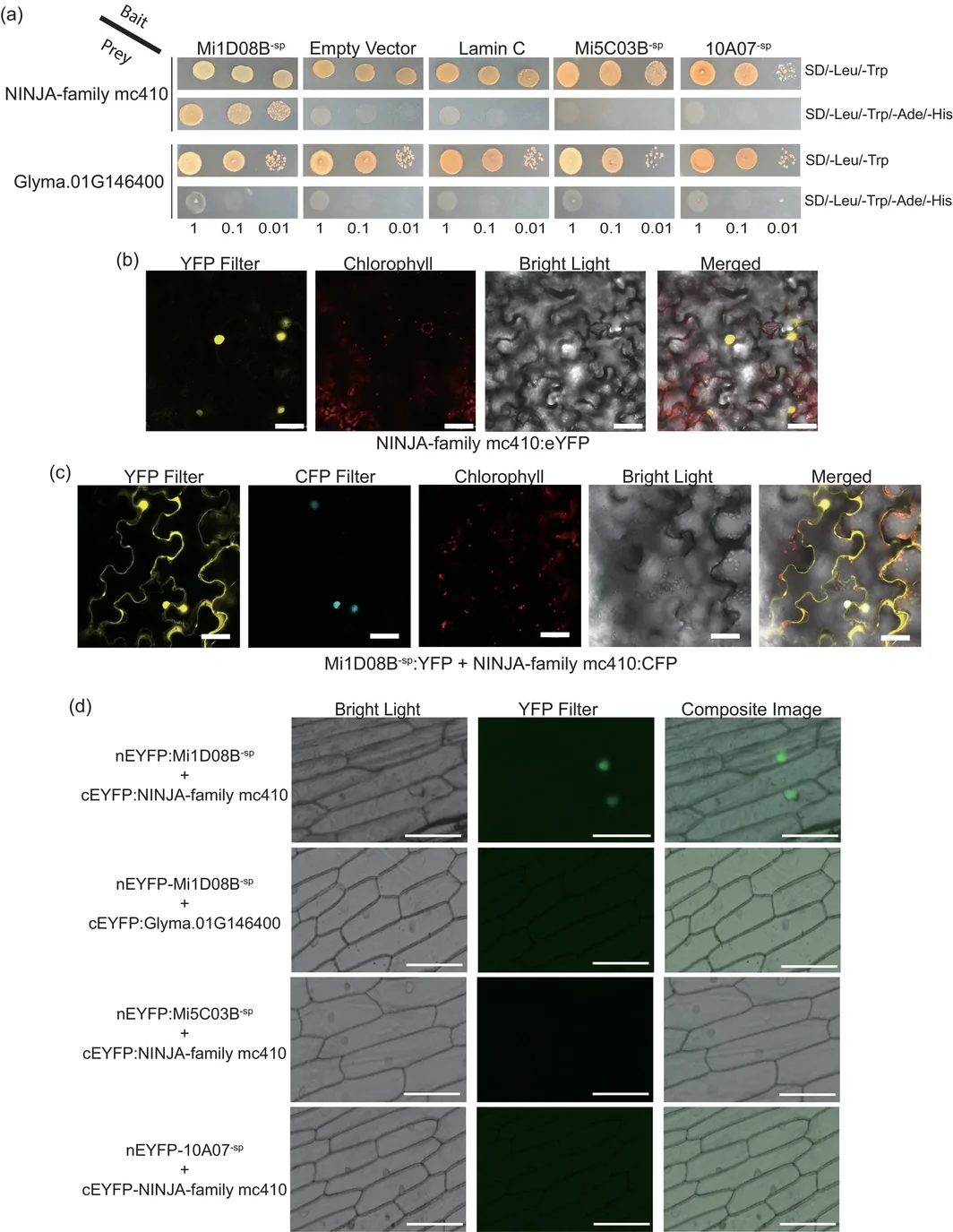
Mi1D08B^‐sp^ interacts with NINJA‐family mc410 in yeast and in planta. (a) Yeast two‐hybrid co‐transformation assay showing specific Mi1D08B–mc410 interaction. Only yeast co‐expressing bait Mi1D08B^‐sp^ and full‐length NINJA‐family mc410 grew on selective SD/−Leu/−Trp/−Ade/−His medium, whereas cells containing negative bait controls (empty vector, human Lamin C, 10A07, Mi5C03B) or a homolog of NINJA‐family mc410 (Glyma.01G146400) did not, indicating a specific Mi1D08B–mc410 interaction. (b) Subcellular localization of mc410:eYFP fusion in *Nicotiana benthamiana* leaf cells. Confocal image showing strong nuclear accumulation of mc410:eYFP, consistent with its identity as a NINJA‐family transcription regulator. (c) Co‐localization of Mi1D08B^‐sp^:eYFP and mc410:eCFP in *N. benthamiana* leaf cells. Merged confocal image shows extensive overlap of YFP and CFP signals in the nucleus when the two proteins are co‐expressed, with no obvious re‐localization upon co‐expression. (d) Bimolecular fluorescence complementation (BiFC) assay in onion epidermal cells showing specific Mi1D08B–mc410 interaction. Co‐expression of nEYFP:Mi1D08B^‐sp^ and cEYFP:mc410 reconstitutes YFP fluorescence in the nucleus, whereas negative combinations (nEYFP:Mi1D08B^‐sp^ + cEYFP:Glyma.01G146400, nEYFP:5C03B^‐sp^ + cEYFP:mc410, and nEYFP:10A07^‐sp^ + cEYFP:mc410) show no signal, confirming the specificity of the interaction. All images are representative of independent replicates; fluorescence channels and scale bars are indicated on the images. Scale Bars: 50 μm (b, c) and 275 μm (d).

To explore the potential function of the *Mi1D08B* effector in host cell physiology, we constructed and screened a gall‐specific yeast two‐hybrid (Y2H) prey library using *Mi1D08B* ^*‐sp*^ was used as a bait. The prey library was prepared RNA isolated from galls collected from *M. incognita*—inoculated Williams 82 at 5‐, 8‐, and 11‐day post‐inoculation (dpi). The final library cell density was 4.35 × 10^7^ colonies per ml, suggesting the high quality of the library. Thirty‐five positive colonies were identified in our initial screen. Of these 35, three independent colonies repeatedly elicited the highest reporter activation. Prey vectors were isolated from these three clones and sequenced, and all were found to correspond to the C‐terminal region of the NINJA‐family gene *mc410* (*Glyma.09G193900*), a transcriptional repressor that functions in the jasmonic acid (JA) signaling pathway. To confirm the specificity of the interaction, yeast co‐transformation assays were conducted. In this assay, the empty bait vector and the bait vectors expressing the human Lamin C, the cyst nematode 10A07, or the *M. incognita* effector Mi5C03B were included as negative controls. Another NINJA‐family gene (*Glyma.01G146400*) was also used as a negative prey control to test for interaction specificity. Only yeast cells co‐expressing Mi1D08B^‐sp^ and the full‐length NINJA‐family protein mc410 grew on the SD/‐Leu/‐Trp/‐Ade/‐His medium, implying a specific interaction between Mi1D08B^‐sp^ and the NINJA‐family mc410 (Figure 3a).

### Mi1D08B ^‐sp^ and NINJA‐family mc410 co‐localize and interact in the plant nucleus

To determine where mc410 acts in plant cells, we expressed an mc410:eYFP fusion in *N. benthamiana* leaves and imaged its fluorescence after agroinfiltration. As expected for a member of the NINJA family, mc410:eYFP was accumulated in the nucleus (Figure 3b). Given that Y2H assays pointed to physical interaction between mc410 and Mi1D08B, we co‐expressed Mi1D08B^‐sp^:eYFP together with mc410:eCFP to test whether the two proteins occupy the same compartment in *planta* and whether co‐expression alters their distribution. Confocal images showed strong overlap of YFP and CFP signals in the nucleus, indicating that Mi1D08B and mc410 co‐localize in the plant nucleus and that their co‐expression does not noticeably change their subcellular localization (Figure 3c). These results support a nuclear site for the interaction observed in the Y2H assay.

We next performed a bimolecular fluorescence complementation (BiFC) assay to confirm Mi1D08B–mc410 interaction in *planta*. Mi1D08B^‐sp^ was fused to the N‐terminal half of YFP (nEYFP:Mi1D08B^‐sp^), while mc410 was fused to the C‐terminal half (cEYFP). The *M. incognita* effector Mi5C03B^‐sp^ and 10A07^‐sp^ effectors fused to nEYFP (nEYFP:10A07^‐sp^ and nEYFP:5C03B^‐sp^) were used as negative controls. In addition, Glyma.01G146400 fused to cEYFP (cEYFP:Glyma.01G146400) was used as negative control. Co‐expression of Mi1D08B^‐sp^ and mc410 in onion epidermal cells reconstituted YFP fluorescence in the nucleus, indicating a specific interaction between the two proteins (Figure 3d). No YFP signal was detected in three negative combinations (nEYFP:Mi1D08B^‐sp^ + cEYFP:Glyma.01G146400, nEYFP:5C03B^‐sp^ + cEYFP:mc410, and nEYFP:10A07^‐sp^ + cEYFP:mc410), confirming the specificity of the Mi1D08B–mc410 interaction.

### *NINJA‐family mc410* is upregulated in giant cells during the mid and late stages of nematode infection

**Figure 4:**
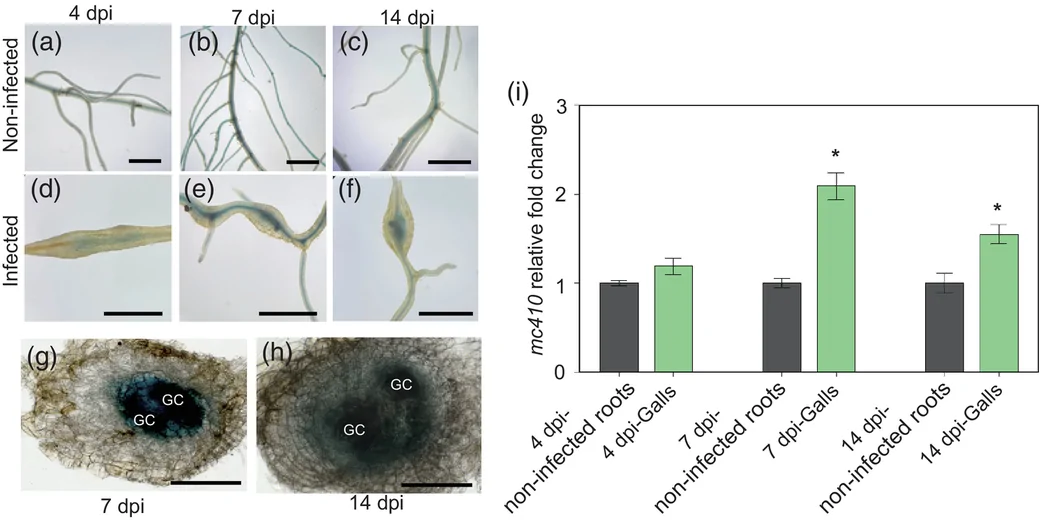
Spatial and temporal induction of *NINJA‐family mc410* during *Meloidogyne incognita* infection. (a–c) GUS histochemical staining of transgenic hairy roots (GFP‐positive transformants) under non‐infected conditions at 4, 7, and 14 dpi, showing promoter activity confined to vascular tissues. (d–f) GUS staining in infected roots at 4 (d), 7 (e), and 14 (f) dpi showing progressive activation of the *mc410* promoter during nematode infection. Weak GUS staining is detected at 4 dpi, whereas strong staining is observed at 7 and 14 dpi. (g–h) Transverse sections of galls at 7 dpi (g) and 14 dpi (h) demonstrating GUS activity localized to giant cells. (i) RT‐qPCR analysis of *mc410* transcript abundance in gall tissues at 4, 7, and 14 dpi relative to non‐infected roots. Bars represent mean ± SE from 3 independent biological replicates. Asterisks indicate statistically significant differences between galls and non‐infected roots (control) (*P* < 0.05, *t*‐test). Scale bars: 750 μm (a–c), 350 μm (d–f), and 275 μm (g and h).

To determine the spatial and temporal expression patterns of *mc410* during M. incognita infection, we generated a β‐glucuronidase (GUS) reporter construct controlled by the *NINJA‐family mc410* promoter and introduced it into transgenic hairy roots, where successful transformation was confirmed by GFP fluorescence. GUS activity was then examined under both non‐infected and *M. incognita*‐infected conditions. In non‐infected roots, GUS activity was observed in vascular root tissues at all time points (Figure 4a–c). In infected roots, weak GUS staining was observed in galls at 4 dpi (Figure 4d), whereas at 7 dpi and 14 dpi strong GUS staining was detected in galls (Figure 4e,f). Cross sections of the 7 and 14 dpi galls revealed GUS activity in giant cells (Figure 4g,h), supporting a possible functional interaction with the nematode effector Mi1D08B in the feeding cells. Consistent with the promoter activity assays, RT‐qPCR analysis showed significant increases in the transcript abundance of *mc410* transcripts in gall tissues at 7 and 14 dpi as compared with non‐infected roots (Figure 4i). Taken together, these results indicate that *mc410* is induced in response to nematode infection, with activation beginning as early as 4 dpi, peaking at 7 dpi, and decreasing slightly thereafter.

### *mc410* functions as a positive regulator of soybean susceptibility to *M. incognita*

**Figure 5:**
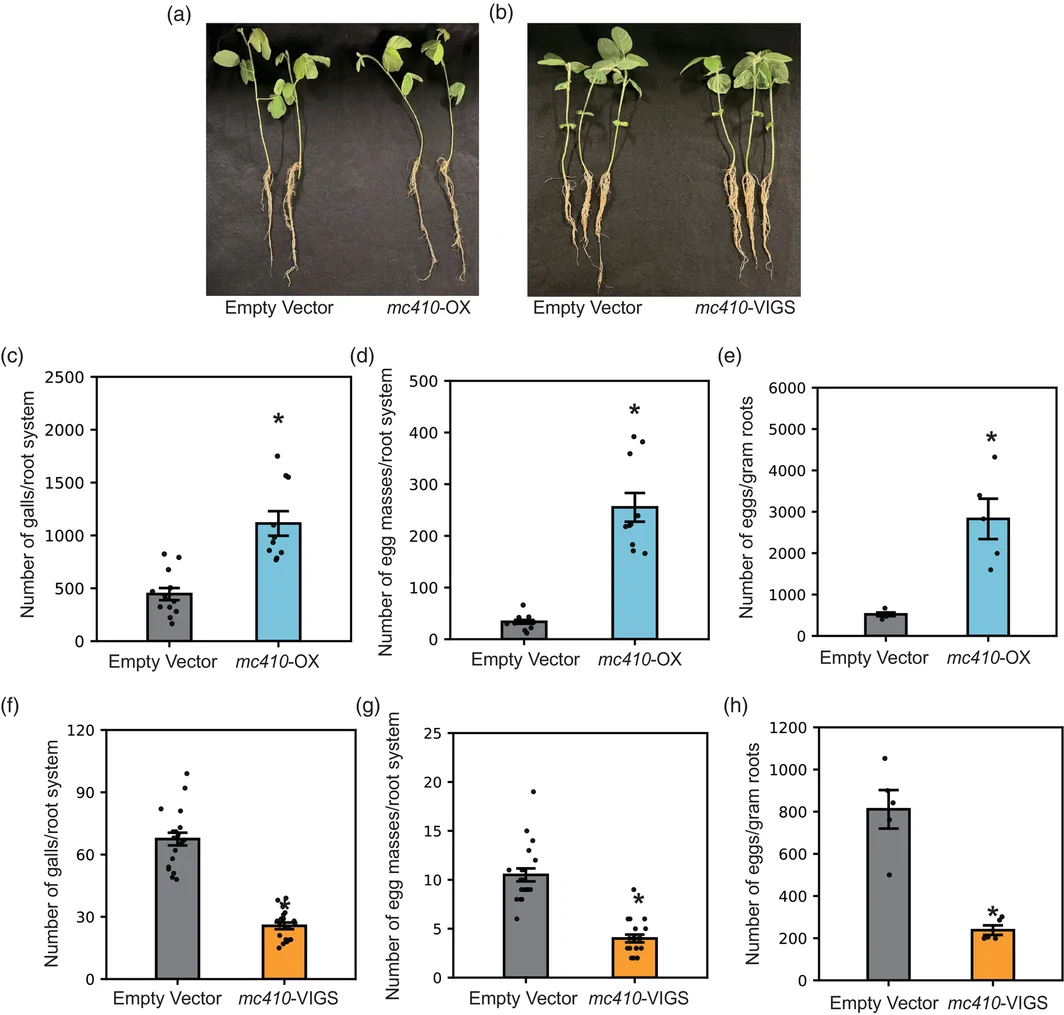
*mc410* promotes soybean susceptibility to *Meloidogyne incognita*. (a, b) Representative images of composite plants with transgenic hairy roots overexpressing *mc410* (a) and *mc410*‐silenced roots (b) alongside their respective vector controls. (c–e) Infection phenotypes of *mc410* overexpression plant (*mc410*‐OX) versus empty vector controls, showing the numbers of galls per plant (c), egg masses per plant (d), and eggs per gram roots (e). Sample sizes: (c, d), empty vector (*n* = 13) and *mc410*‐OX (*n* = 10); (e) *n* = 5 per group. (f–h) Infection phenotypes for *mc410*‐silenced (*mc410*‐VIGS) plants versus TRV2 control plants, showing the numbers of galls per plant (f), egg masses per plant (g), and eggs per gram roots (h). Dots represent individual biological replicates; bars indicate mean ± SE. Asterisks denote statistically significant differences between *Mi1D08B*‐OX or *mc410*‐VIGS plants and the corresponding vector control plants (*P* < 0.05, *t*‐test). Sample sizes: (f, g), *n* = 20 per group; (h) *n* = 5 per group. Raw data for gall numbers, egg mass numbers, and eggs per gram of root tissues in *mc410*‐overexpressing, *mc410*‐silenced, and control plants are provided in Table S1.

To examine the functional role of mc410 in soybean *M. incognita* interaction*s*, we manipulated the expression of *mc410* using overexpression and virus‐induced gene silencing (VIGS) approaches. Overexpression of *mc410* in transgenic hairy roots achieved approximately 15‐fold increases in randomly collected root samples (Figure S1B), whereas TRV vector‐mediated gene silencing achieved approximately 55% downregulation (Figure S1C). No visible morphological differences were detected in overexpressing or silenced plants when compared with the corresponding control (Figure 5a,b). Remarkably, overexpression of *mc410* increased soybean susceptibility to *M. incognita* as compared with the empty vector control (Figure 5c–e). Specifically, overexpression of *mc410* resulted in 150% increases in the number of galls per plant, a 659% increase in the number of egg masses per plant, and a 442% increase in the number of eggs per gram roots (Figure 5c–e). In contrast to overexpression, silenced plants showed considerable decreases in plant susceptibility with a 62% reduction in the number of galls, a 62% reduction in the number of egg masses, and a 71% reduction in egg production (Figure 5f–h). Together, these results imply that *mc410* functions as a positive regulator of soybean susceptibility to *M. incognita*.

### Mi1D08B disrupts JA biosynthesis and signaling by creating a bottleneck at the OPDA‐to‐JA conversion step

**Figure 6:**
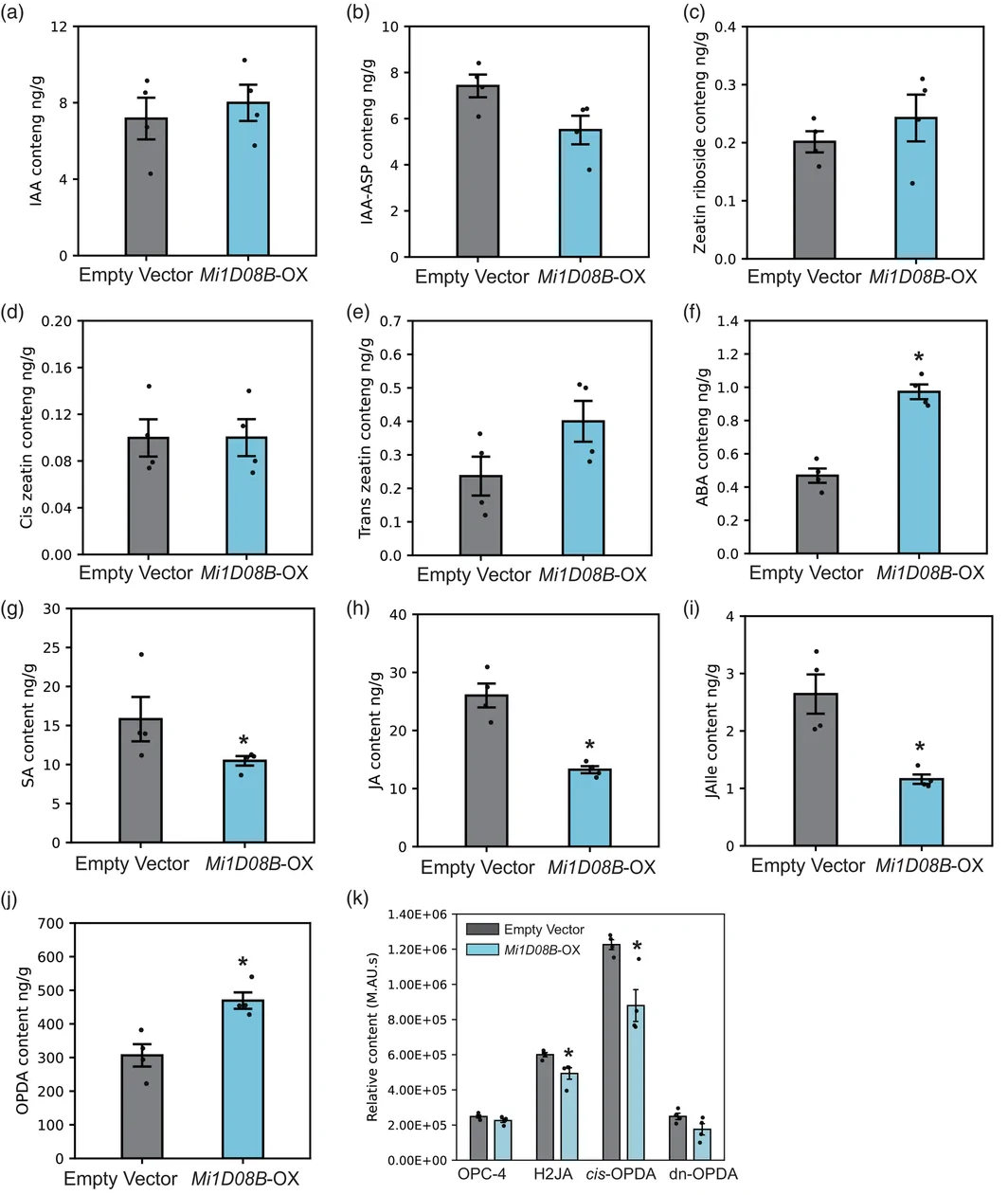
Mi1D08B alters defense‐related hormone accumulation in soybean roots. (a–e) Levels of auxin, including indole‐3‐acetic acid (IAA, a) and its conjugate indole‐3‐acetic acid–aspartate (IAA‐Asp, b), and cytokinins, including zeatin riboside (Zr, c), *cis*‐zeatin (c‐Z, d), and *trans*‐zeatin (t‐Z, e), show no significant differences between *Mi1D08B*‐overexpressing and control root samples. (f) Level of Abscisic acid (ABA) is increased in *Mi1D08B*‐overexpressing roots relative to control. (g–i) Levels of the defense hormones salicylic acid (SA, g), jasmonic acid (JA, h), and the bioactive jasmonate conjugate JA‐Ile (i) are significantly decreased in *Mi1D08B*‐overexpressing roots. (j) Level JA precursor 12‐oxo‐phytodienoic acid (12‐OPDA) accumulates to higher levels in *Mi1D08B*‐overexpressing roots. Data were obtained from four biological replicates. Dots represent individual replicates, and bars indicate the mean ± SE. Asterisks denote statistically significant differences between *Mi1D08B‐OX* and the corresponding vector control plants (*P* < 0.05, *t*‐test). (k) Quantification of jasmonate‐related metabolites associated with the OPDA‐to‐JA conversion pathway in *Mi1D08B*‐overexpressing soybean roots. Levels of 3‐oxo‐2‐(2‐(Z)‐pentenyl)cyclopentane‐1‐butyric acid (OPC‐4), 9,10‐dihydrojasmonic acid (H2JA), *cis*‐12‐oxo‐phytodienoic acid (*cis*‐OPDA), and dinor‐12‐oxo‐phytodienoic acid (dn‐OPDA) were determined by LC–MS/MS in *Mi1D08B*‐overexpressing and control soybean hairy roots. Bars represent mean ± SE. Statistical significance was determined using *t*‐test (*P* < 0.05). Sample size: *n* = 4 biological replicates per treatment. Each dot represents an individual biological replicate.

Because NINJA‐family proteins are central regulators of jasmonic acid (JA) signaling (Pauwels et al., 2010; Zhou et al., 2023), overexpression of *Mi1D08B* would be expected to alter JA biosynthesis and signaling in soybean roots if its function is mediated through interaction with the NINJA‐family protein mc410. Hormone quantification revealed that levels of auxin [Indole‐3‐acetic acid (IAA) and aspartate conjugate of IAA (IAA‐Asp)] (Figure 6a,b) and cytokinins [Zeatin riboside (Zr), *cis*‐Zeatin (c‐Z), and *trans*‐Zeatin (t‐Z)] (Figure 6c–e) were comparable between *Mi1D08B*‐overexpressing and control plants. In contrast, the levels of abscisic acid (ABA) (Figure 6f) and defense‐related hormones, including salicylic acid (SA) (Figure 6g), JA (Figure 6h), and the bioactive conjugate Jasmonoyl‐L‐isoleucine (JA‐Ile) (Figure 6i), were significantly reduced in *Mi1D08B*‐overexpressing roots compared with control. Notably, despite the reduced accumulation of JA and JA‐Ile, the JA precursor 12‐oxo‐phytodienoic acid (12‐OPDA) accumulated to a significantly higher level in *Mi1D08B*‐overexpressing roots (Figure 6j).

To further investigate how Mi1D08B affects jasmonate metabolism, we quantified additional metabolites that represent key intermediates and products of the OPDA‐to‐JA conversion pathway. These metabolites provide insight into the metabolic flux through the peroxisomal phase of JA biosynthesis and help distinguish between alterations in OPDA production and downstream JA‐generating reactions. In *Mi1D08B*‐overexpressing roots, levels of several jasmonate‐related metabolites were reduced relative to control plants. Specifically, 3‐oxo‐2‐(2‐(Z)‐pentenyl)cyclopentane‐1‐butyric acid (OPC‐4), 9,10‐dihydrojasmonic acid (H2JA), cis‐12‐oxo‐phytodienoic acid (*cis*‐OPDA), and dinor‐12‐oxo‐phytodienoic acid (dn‐OPDA) decreased by approximately 10%, 18%, 29%, and 30%, respectively (Figure 6k). Together, these data indicate that *Mi1D08B* overexpression strongly suppresses JA accumulation and is associated with altered levels of multiple metabolites linked to the peroxisomal conversion of OPDA to JA, further supporting the existence of a bottleneck in this branch of the jasmonate biosynthetic pathway. Together, these data indicate that *Mi1D08B* overexpression strongly suppresses JA accumulation and is associated with altered levels of multiple metabolites linked to the peroxisomal conversion of OPDA to JA, supporting the existence of a bottleneck in this branch of the jasmonate biosynthetic pathway. Given the central role of JA and JA‐Ile in activating defense responses, these findings point to a corresponding reduction in JA‐mediated defense signaling.

**Figure 7:**
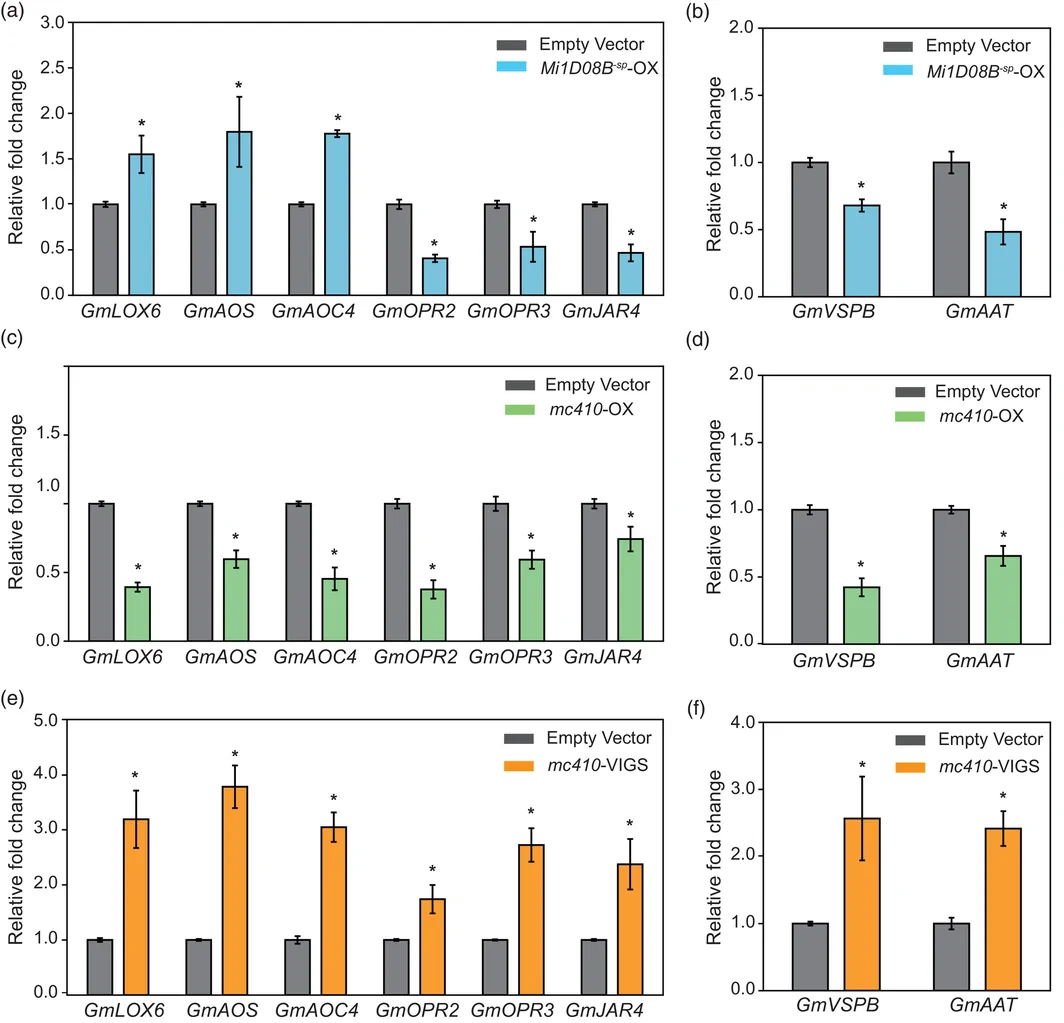
Expression profiling of jasmonic acid biosynthetic and responsive genes in *Mi1D08B*‐overexpressing, *mc410*‐overexpressing, and *mc410*‐silenced soybean roots. (a) RT‐qPCR analysis of OPDA‐biosynthetic and OPDA‐reduction genes in *Mi1D08B*‐overexpressing roots relative to controls. The OPDA‐biosynthetic genes *GmLOX6* (*13‐lipoxygenase*), *GmAOS* (*allene oxide synthase*), and GmAOC4 (*allene oxide cyclase 4*) were upregulated, whereas OPDA‐reductases *GmOPR2* and *GmOPR3* and the JA‐conjugation enzyme *JAR4* were downregulated relative to empty vector control. (b) RT‐qPCR analysis of JA marker genes in *Mi1D08B*‐overexpressing roots. JA‐responsive marker genes *GmVSP‐B* (*Vegetative Storage Protein B*) and *GmAAT* (*Amino Acid Transporter*) were downregulated in *Mi1D08B*‐overexpressing roots relative to empty vector control. (c, d) RT‐qPCR analysis of JA‐biosynthetic (c) and JA‐responsive genes (d) in *mc410* overexpression plants showing downregulation relative to empty vector control. (e, f) RT‐qPCR analysis of JA‐biosynthetic (e) and JA‐responsive genes (f) in *mc410* silenced plants showing upregulation relative to empty vector control. Bars represent mean ± SE from three independent biological replicates of root tissues, each analyzed with three technical replicates. Relative fold‐change values indicate gene expression levels in *Mi1D08B*‐overexpressing, *mc410*‐overexpressing, or *mc410*‐silenced plants compared with the corresponding empty vector controls, which were set to 1. Asterisks indicate statistically significant differences from the empty vector controls as determined by *t*‐tests (*P* < 0.05).

To examine this hypothesis, we quantified the transcript levels of key JA‐biosynthetic and JA‐responsive genes in *Mi1D08B*‐overexpressing, *mc410*‐overexpressing, and *mc410*‐silenced roots relative to non‐infected control samples. In *Mi1D08B*‐overexpressing roots, genes encoding plastid‐localized enzymes that produce the immediate precursor of JA (*cis*‐12‐oxo‐phytodienoic acid, OPDA), including *13‐lipoxygenase* (*GmLOX6*), *allene oxide synthase* (*GmAOS*), and *allene oxide cyclase 4* (*GmAOC4*), were upregulated (Figure 7a). In contrast, the expression of *12‐oxo‐phytodienoate reductase 2* (*GmOPR2*) and *GmOPR3*, which catalyze OPDA conversion to JA, was downregulated (Figure 7a). Consistent with reduced JA signaling, *jasmonic resistant 4* (*JAR4*), which is involved in the conversion of JA to JA‐Ile, the bioactive form of JA, was downregulated (Figure 7a). The JA marker genes *Vegetative Storage Protein B* (*GmVSP‐B*) and *Amino Acid Transporter* (*GmAAT*) were also downregulated in *Mi1D08B*‐overexpressing roots (Figure 7b). Notably, *mc410* overexpression and silencing produced complementary effects. *mc410* overexpression showed reduced expression of JA and SA biosynthesis/signaling genes (Figure 7c,d), whereas *mc410* silencing led to upregulation of the same gene set (Figure 7e,f).

Because SA levels were reduced in *Mi1D08B*‐overexpressing roots, we quantified the transcript abundance of *PR1* (pathogenesis‐related protein 1) and *PR2*, two canonical SA‐responsive defense marker genes. Consistent with the reduced SA accumulation, *PR1* and *PR2* expression levels were significantly decreased in *Mi1D08B*‐overexpressing roots (Figure S2A). A similar reduction in *PR1* and *PR2* transcript levels was observed in *mc410*‐overexpressing roots, whereas their expression was significantly increased in mc410‐silenced roots (Figure S2B,C). Together, the coordinated upregulation of OPDA‐biosynthetic genes and downregulation of OPDA‐reduction, JA‐conjugation, and JA‐responsive genes indicate that Mi1D08B creates a bottleneck at the OPDA‐to‐JA conversion step, thereby suppressing JA‐mediated defense signaling to facilitate *M. incognita* infection. In addition, the reduced accumulation of SA, together with the decreased expression of the SA marker genes *PR1* and *PR2*, suggests that Mi1D08B indirectly suppresses SA‐dependent defense responses, likely as a consequence of its modulation of JA biosynthesis and signaling through the mc410 regulatory pathway.

## DISCUSSION

Mi1D08B is a broadly conserved effector across the RKN genus, with clear homologs in *M. enterolobii*, *M. hapla*, and *M. graminicola*. Such cross‐species conservation is characteristic of core parasitism effectors with essential functions during infection, as reported for other conserved RKN effector families such as CRTs and CM proteins (Feng et al., 2024; Liu et al., 2024). Sequence analysis also shows that Mi1D08B effector is a cysteine‐enriched effector, containing ten cysteine residues, a feature commonly associated with virulence effectors across diverse phytopathogens (Duplessis et al., 2011; Saunders et al., 2012; Zhang et al., 2017). The cysteines are not randomly distributed and include clusters (e.g., CPTNICICG, and CTSCANFKQLTC). Cysteine‐rich domains are thought to enhance protein stability and functionality in the host environment, often through the formation of disulfide bonds (Saunders et al., 2012; Sevier & Kaiser, 2002; Wang et al., 2020). Similar cysteine‐rich effectors have been described in plant‐parasitic nematodes, including MiSGCR1 effector from *M. incognita*, which suppresses host cell death triggered by pathogen infection (Nguyen et al., 2018).

Numerous nematode effectors are targeted to the host nucleus via canonical nuclear localization signals to reprogram host transcription and splicing (Chen et al., 2017; Hewezi, 2024; Hewezi et al., 2015; Shi, Mao, Zhang, Zhang, et al., 2018; Truong et al., 2021; Zhao et al., 2024). However, Mi1D08B accumulates in the plant nucleus despite lacking an obvious classical NLS. This likely occurs through exploiting non‐classical, importin‐independent nuclear import mechanisms. Several plant pathogen effectors, including the *Phytophthora infestans* RXLR effectors Pi04314 (Boevink et al., 2016) and some oomycete CRN family members (Schornack et al., 2010), as well as the *Ustilago maydis* effector See1 (Redkar et al., 2015), have been shown to accumulate in the host nucleus despite lacking clear canonical nuclear localization signals. Another possibility is passive diffusion, as small proteins can pass through nuclear pore complexes without a classical import signal. Mi1D08B may also enter the nucleus during transient remodeling of the nuclear envelope, allowing access to the nucleoplasm independent of active nuclear import.

Consistent with the virulent function of nematode effectors, *Mi1D08B* ^*‐sp*^ overexpression increased the numbers of galls, egg masses, and egg production (Figure 2b–d). This pathogenicity function appears to be mediated through interaction with the soybean NINJA‐family mc410, a key negative regulator of JA signaling. Our data suggest that *Mi1D08B*–mc410 interaction enhances or stabilizes mc410 to maintain its repression function, thereby inhibiting JA‐mediated defense responses. Similarly, the *M. incognita* effector Mi2G02 stabilizes the transcription factor GT‐3a by suppressing its degradation through the 26S proteasome pathway, maintaining GT‐3a at high levels to repress the growth regulator genes TOZ and RAD23c (Zhao et al., 2024). This suggestion is supported by our data showing that overexpression of *mc410* mimics *Mi1D08B* overexpression of increased plant susceptibility along with downregulation of JA‐dependent defense genes. The opposite phenotype of reduced nematode susceptibility and upregulation of JA‐mediated defense gene expression observed in *mc410*‐silenced plants further supports this scenario.

Considering the well‐established role of NINJA proteins in the JA signaling pathway (Pauwels et al., 2010; Zhou et al., 2023), the significant decreases in JA and JA‐Ile levels in transgenic roots overexpressing *Mi1D08B* (Figure 6h,i) provide strong functional support for the biological relevance of the Mi1D08B–mc410 interaction. The consistency between the biochemical interaction and the downstream shift in jasmonate accumulation strongly argues that mc410 is a bona fide host target of Mi1D08B rather than a nonspecific or an incidental interactor. Disruption of mc410 function by Mi1D08B likely contributes directly to the observed suppression of JA homeostasis, linking effector binding to measurable hormonal outcomes in planta. Importantly, gene expression data showing elevated expression of plastidial OPDA‐biosynthesis genes concurrent with decreased expression of peroxisomal reduction, JA‐conjugation, and JA‐responsive genes in *Mi1D08B*‐overexpression roots (Figure 7a,b) are most consistent with a metabolic bottleneck at the OPDA to JA conversion step. The increased expression of OPDA‐biosynthesis genes may also represent an indirect compensatory response to reduced JA levels or weakened JA signaling. Additionally, the accumulation of OPDA may reflect not only transcriptional repression of OPR genes, but also additional mechanisms such as altered peroxisomal import of OPDA or impaired downstream β‐oxidation steps. Together with the observed increase in total OPDA level and the concomitant decrease in JA/JA‐Ile levels, these results suggest that Mi1D08B binding to mc410 interferes with the peroxisomal conversion of OPDA to JA. This disruption likely limits JA biosynthesis, thereby reducing JA accumulation and weakening downstream JA‐mediated defense responses. Because JA‐mediated defenses play a critical role in restricting nematode infection (Shi et al., 2025; Shi, Mao, Zhang, Ling, et al., 2018; Shi, Mao, Zhang, Zhang, et al., 2018; Verhoeven et al., 2023), the ability of Mi1D08B to associate with mc410 and reduce JA and JA‐Ile levels provides a mechanistic explanation for how this effector enhances host susceptibility and supports nematode pathogenicity.

The altered levels of additional metabolites further support a defect in the peroxisomal phase of jasmonate metabolism. In particular, the coordinated reductions in OPC‐4, H2JA, *cis*‐OPDA, and dn‐OPDA (Figure 6k) are consistent with reduced metabolic flux through the OPDA‐to‐JA branch. Although the decreases in OPC‐4 and dn‐OPDA were modest, their coordinated reduction with JA and JA‐Ile suggests that *Mi1D08B* overexpression broadly perturbs jasmonate metabolism rather than affecting only a single intermediate. Together, these metabolite changes support the conclusion that Mi1D08B interferes with peroxisomal jasmonate biosynthesis downstream of OPDA accumulation.

Genetic evidence from Arabidopsis provides further support for the importance of OPDA to nematode resistance. For example, mutants that disrupt early OPDA production are hyper‐susceptible to *M. hapla*, and loss of OPDA perception also increases galling (Gleason et al., 2016). In contrast, some downstream JA‐biosynthetic mutants show less dramatic galling phenotypes, indicating that intact OPDA synthesis and/or perception is particularly important for maintaining basal resistance to RKN. These findings are consistent with our biochemical and nematode susceptibility data, indicating that perturbation of OPDA biosynthesis or perception, as well as impaired OPDA‐to‐JA conversion, either genetically or through Mi1D08B‐mediated interference with mc410 function, enhances nematode parasitism.

It may be important to mention that the dorsal‐gland effector MiMSP32 from *M. incognita* also modulates host jasmonate biology but acts at a distinct node of the oxylipin pathway. MiMSP32 physically interacts with host OPR2 and promotes susceptibility when overexpressed or when OPR2 function is lost, indicating that MiMSP32 enhances parasitism by targeting the OPDA to JA conversion pathway (Verhoeven et al., 2023). Unlike Mi1D08B, which acts in the nucleus by modulating the function of the NINJA‐family co‐repressor mc410 and thereby imposing a peroxisome‐linked metabolic bottleneck that lowers JA/JA‐Ile, MiMSP32 binds a cytosolic/peroxisome‐associated enzyme (OPR2) and tends to increase 12‐OPDA without producing a strong change in total JA or JA‐Ile when overexpressed in tomato plants. It appears that both effectors converge on diminishing effective JA‐mediated anti‐nematode responses and increasing host susceptibility, but they differ in molecular entry point (modulation of a nuclear co‐repressor versus direct targeting of a 12‐OPDA reductase) and in the biochemical signature of hormone changes (clear JA/JA‐Ile reduction for Mi1D08B versus altered 12‐OPDA with no large JA change for MiMSP32). In addition, the nuclear‐localized effector MiISE23 binds multiple JAZ repressors and competes with COI1 for the Jas domain, thereby stabilizing JAZ proteins and blocking JA‐Ile–mediated JAZ degradation (Shi et al., 2025). This leads to suppression of JA‐responsive genes, reduction of JA‐Ile level, and increases susceptibility to *M. incognita* (Shi et al., 2025). These complementary strategies, including nuclear repression of JA signaling mediated by Mi1D08B, direct modulation of OPDA metabolism mediated by MiMSP32, and blockage of JA signaling at the level of hormone perception mediated by MiISE23 demonstrate that RKNs employ multiple, non‐redundant tactics to subvert oxylipin‐based defenses, and suggest that simultaneous targeting of nuclear regulators and metabolic enzymes could produce additive effects on susceptibility.

JA and SA pathways typically exhibit antagonistic relationships, with their activation often occurring through mutual negative regulation as a defense strategy (Kunkel & Brooks, 2002; Sato et al., 2010; Thaler et al., 2012). Several nematode effectors have been shown to specifically interfere with JA signaling, such as MiISE6, MiISE23, and Mj2G02 (Shi et al., 2025; Shi, Mao, Zhang, Zhang, et al., 2018; Song et al., 2021). In MiPDI1‐overexpression lines, the JA marker gene *PDF1.2a* was strongly induced whereas the SA marker *AtPR1a* was reduced. On the contrary, in the *sap12* mutant, an interactor of *MiPDI1*, both *PDF1.2a* and *PR1a* showed elevated expression (Zhao et al., 2020). These observations deviate from the classical JA–SA antagonism model. A similar pattern is observed in the case of *MiEFF1*, whose interactors AtGAPC1 and AtGAPC2 also influence hormone signaling: *PR1a* and *PDF1.2a* were both upregulated in the corresponding mutants (Truong et al., 2021). In this study, overexpression of the *Mi1D08B* effector led to simultaneous decreases in JA and SA levels (Figure 6g,h). This pattern suggests that Mi1D08B does not adjust the balance between the two pathways but instead targets a regulatory node that affects both JA and SA. Because NINJA‐family proteins can recruit the TPL co‐repressor complex to broadly modulate immune‐related transcription (Pauwels et al., 2010), we propose that the Mi1D08B–mc410 interaction reinforces this repression and thereby dampens JA signaling. The observed reduction in SA levels may not result from direct targeting of the SA pathway by Mi1D08B but could instead be an indirect consequence of altered JA signaling and the extensive cross talk that exists between these hormone networks. As a result, such reinforcement would not only inhibit JA downstream activation but also attenuate SA accumulation or signaling output. This mechanism reveals another strategy used by RKN to suppress host immunity and offers new perspectives on the coordination among plant hormone pathways.

**Figure 8:**
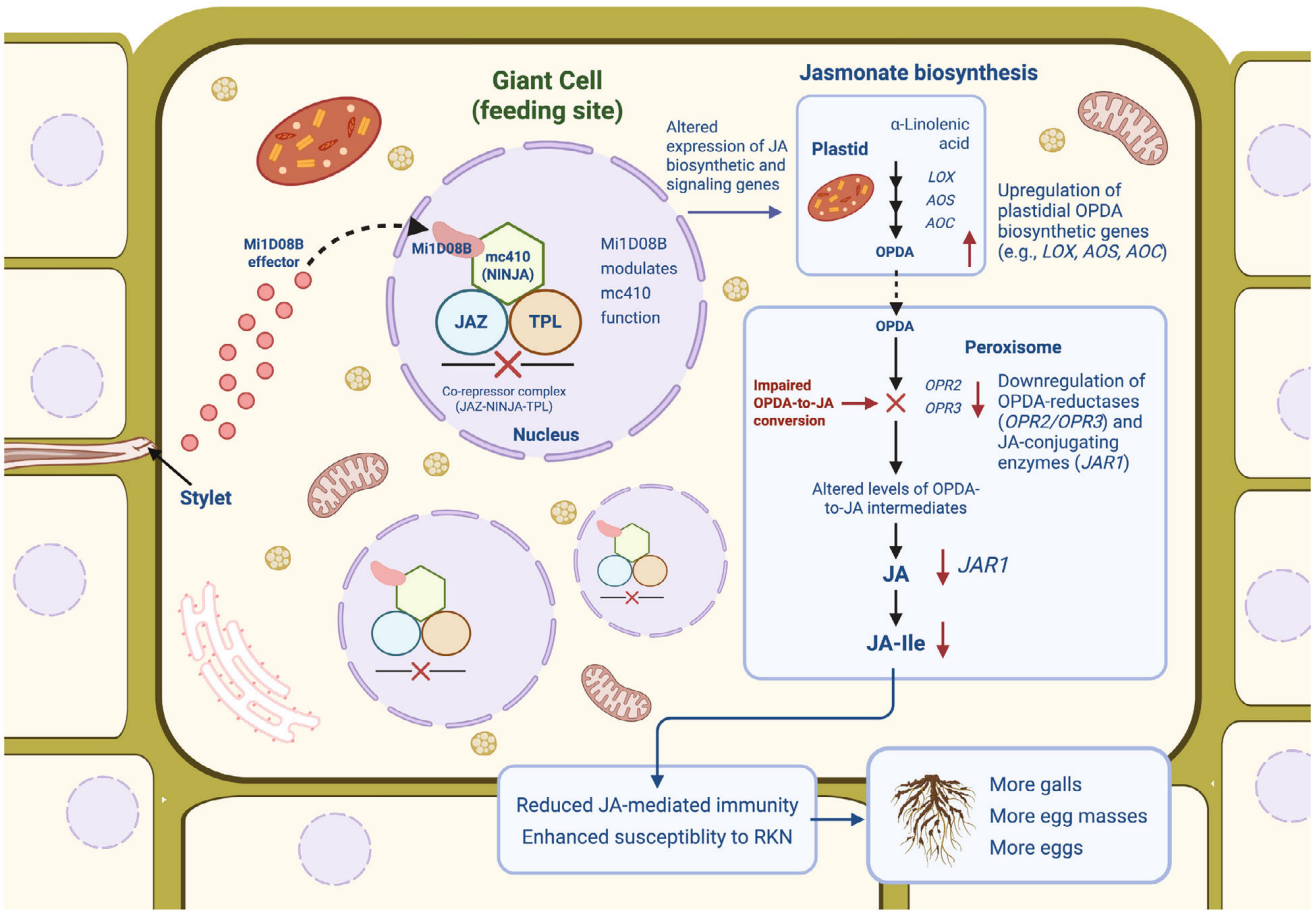
Working model of Mi1D08B‐mediated suppression of jasmonate‐dependent defense responses to promote *M. incognita* susceptibility. After secretion into plant cells, Mi1D08B localizes to the nucleus, where it physically interacts with the NINJA‐family transcriptional repressor mc410 and alters its activity. This interaction is associated with increased transcript levels of plastid‐localized OPDA‐biosynthetic genes (*GmLOX*6, *GmAOS*, and *GmAOC4*), decreased expression of genes involved in OPDA reduction (*GmOPR2* and *GmOPR3*). As a result, the jasmonate precursor 12‐OPDA accumulates, whereas the levels of downstream jasmonate metabolites, including JA and its bioactive conjugate JA‐Ile, decrease. This suggests a bottleneck at the OPDA‐to‐JA conversion step, leading to attenuation of JA‐mediated defense signaling and ultimately promoting gall formation and nematode reproduction. Arrows indicate activation or metabolic progression, whereas X symbols indicate inhibition of gene expression, metabolic conversion, or defense signaling. The illustration was generated using BioRender.

In conclusion, our results establish a new paradigm for effector‐mediated hormone suppression in which a nuclear‐localized nematode effector targets a NINJA‐family regulator to impose a metabolic blockage in the peroxisomal OPDA to JA conversion, thereby directly coupling nuclear repression to diminish jasmonate biosynthesis, and JA‐dependent defense response, leading to successful parasitism of soybean plants (Figure 8). This represents, to our knowledge, the first demonstration that an RKN effector rewires hormone production by manipulating a nuclear co‐repressor to effect change in peroxisomal metabolism, a strategy that defines how nematode effectors can modulate host physiology. Beyond its conceptual impact, this mechanism identifies discrete molecular vulnerabilities such as the Mi1D08B–mc410 interaction interface and the downstream peroxisomal steps of jasmonate biosynthesis that could be targeted to restore JA homeostasis during RKN infection to enhance plant resistance.

## MATERIAL AND METHODS

### Plant materials and growth conditions

In all experiments, soybean (*Glycine max*) cultivar Williams 82 seedlings were used. Plants were grown under long‐day conditions (16 h light/8 h dark) at 26°C with 65% relative humidity in a controlled growth chamber.

### Construction of gall‐specific yeast two‐hybrid prey library

A gall‐specific prey library was constructed following the *Make Your Own* “*Mate & Plate™*” *Library System* User Manual (Takara), using the Takara In‐Fusion® SMARTer® Directional cDNA Library Construction Kit. Four‐day‐old seedlings of the susceptible soybean cultivar Williams 82 were inoculated with approximately 500 second‐stage juveniles of *Meloidogyne incognita*. Galls were collected at 5‐, 8‐, and 11‐day post‐inoculation (dpi) for RNA extraction. Approximately 800 galls were collected from at least 50 plants at each time point. Total RNA extracted from the three time points was combined, and 6 μg mRNA was isolated from the combined total of 93.8 μg RNA. cDNA was synthesized from mRNA using SMART III‐modified oligo and SMART MMLV reverse transcriptase (Takara Bio). The synthesized cDNA was amplified with Long Distance PCR (LD‐PCR) using Advantage 2 Polymerase Mix (Takara Bio) with 21 cycles. The PCR product was purified using CHROMA SPIN+TE‐400 Columns (Takara Bio) that select DNA molecules greater than 200 bp. The yeast two‐hybrid prey library was prepared following the library‐scale transformation (*Yeastmaker Yeast Transformation System 2* User Manual). In brief, competent yeast (*Saccharomyces cerevisiae*) cells (strain *Y187*) were transformed with approximately 10 μg cDNA and 3 μg of the prey vector pGADT7‐Rec. The transformed cells were plated on the SD/−leucine medium and incubated at 30°C for 4 days. Then, transformed yeast colonies were collected using liquid freezing medium.

### Yeast two‐hybrid screens and yeast co‐transformation assays

Yeast two‐hybrid (Y2H) screening was performed following the instructions of the *Matchmaker® Gold Yeast Two‐Hybrid System* user manual (Takara). The coding sequence of *Mi1D08B* ^*‐sp*^ (lacking the signal peptide) was amplified by PCR and fused to the DNA activation domain (AD) in the pGBKT7 bait vector. The resulting bait construct was verified by sequencing and then transformed into *Saccharomyces cerevisiae* strain Y2HGold to generate the bait strain, which was selected on SD/−Trp medium. The bait strain was subsequently mated with the RKN gall‐specific prey library to identify interacting proteins. Positive colonies were selected on synthetic dropout (SD/‐Leu/‐Trp/‐Ade/‐His) medium containing the *MEL1* reporter gene encoding *X‐*α‐galactosidase. Primers used for cloning are listed in Table S2.

Specificity of the interaction between Mi1D08B (bait) and NINJA‐family mc410 (prey) was confirmed in yeast co‐transformation assays of bait and prey plasmids in the yeast Gold strain as previously described (Hewezi et al., 2015). In these assays, four different bait vectors were used as negative controls, including the empty vector, bait vectors containing the Human Lamin C or the RKN effector protein *Mi5C03B* (*AY422832*), and the soybean cyst nematode effector 10A07 (Hewezi et al., 2015; Huang et al., 2004). In addition, another NINJA‐family gene *Glyma.01G146400* was used as a negative control and tested for potential interaction with Mi1D08B^*‐sp*^. All transformant cells were plated both on double dropout (SD/‐Leu/‐Trp) and quadruple dropout (SD/‐Ade/‐His/‐Leu/‐Trp) media to assess interaction‐dependent growth. Primers used for cloning are listed in Table S2.

### Bimolecular fluorescence complementation (BiFC) assays

Bimolecular fluorescence complementation (BiFC) assay was conducted as recently described by Hawk et al. (2026). *Mi1D08B* ^*‐sp*^ and *NINJA‐family mc410* were cloned into pSAT4‐nEYFP‐C1 (E81) and pSAT4‐cEYFP‐C1(B) (E82) vectors, respectively, and verified by sequencing. The resulting fusion proteins were transiently co‐expressed in green onion epidermal cells using biolistic bombardment previously described in Hewezi et al. (2008). The nucleus‐localized RKN effector, *5C03B* ^*‐sp*^, and the cyst nematode 10A07 effector were cloned into pSAT4‐nEYFP‐C1 and used as a negative control. Another *NINJA*‐family gene *Glyma.01G146400* was cloned into pSAT4‐cEYFP‐C1(B) and used as another negative control. Onion peels were incubated at 25°C in the dark for 16–24 h, and YFP fluorescence was visualized using the EVOS FL Auto Cell Imaging System (Life Technologies) equipped with a YFP filter. Primers used for cloning are listed in Table S2.

### Overexpression *Mi1D08B* ^*‐sp*^ and its interactor *NINJA‐family mc410* in soybean composite plants

Overexpression constructs for *Mi1D08B* and its interactor, *NINJA*‐family protein *mc410* (*Glyma.09G193900*), were generated. The coding sequences of *mc410* and *Mi1D08B* ^*‐sp*^ were amplified and cloned into the pG2RNAi2 vector containing a GFP reporter gene using the restriction enzymes *AscI* and *AvrII*. Primers used for cloning are listed in Table S2. An empty pG2RNAi2 vector was used as a negative control. Both constructs were sequence‐verified and transformed into *Agrobacterium rhizogenes* strain *K599* to generate composite plants with transgenic hairy roots. *A. rhizogenes* cultures containing the binary vectors or empty vector (control) were injected into the hypocotyls of four‐day‐old soybean seedlings to induce transgenic hairy roots as previously described (Hawk et al., 2026; Sultana et al., 2024). Three‐week post‐inoculation, hairy roots were screened for GFP fluorescence using a fluorescence stereomicroscope. Only roots exhibiting strong, uniform, and homogeneous GFP expression throughout the root tissue were selected for downstream analyses, whereas non‐transgenic roots and roots showing weak or patchy GFP signals were excluded. During the GFP screening, three independent biological samples of GFP‐expressing roots were collected from each treatment for gene expression analysis. The composite plants with transgenic hairy roots were planted in commercial potting soil mix and were then inoculated with *M. incognita* freshly hatched juveniles as mentioned below.

### Silencing *NINJA‐family mc410* using tobacco rattle virus (TRV)‐based VIGS

TRV‐derived TRV1 and TRV2 vectors (Ratcliff et al., 2001) were used to silence *mc410* expression was silenced as recently described (Hawk et al., 2026; Sultana et al., 2024). In brief, a 350 bp fragment of the *mc410* coding sequence was cloned into the pTRV2 vector. The resulting construct was transformed into *Agrobacterium tumefaciens* strain *LBA4404*. Four‐day‐old soybean roots were infiltrated with *A. tumefaciens* containing pTRV1 and pTRV2‐*mc410*. *Agrobacterium* cultures containing pTRV1 and the empty pTRV2 vectors were also infiltrated and used as control. Fourteen‐day post‐infiltration, the plants were planted in commercial potting soil mix and were then inoculated with 500 *M. incognita* juveniles as mentioned below. Meanwhile, three independent biological samples of infiltrated roots (both treatment and control) were collected to determine gene silencing levels using RT‐qPCR. Primer sequences for constructing pTRV2‐*mc410* vector are provided in Table S2.

### Nematode infection assays

Overexpression and silenced plants were inoculated with freshly hatched second‐stage juveniles (J2s) of *M. incognita*. The infection assays were arranged in a randomized complete block design experiment with at least 10 replicated plants. Approximately 5 week post‐inoculation, nematode susceptibility levels were determined using three parameters, including the number of galls per plant, the number of egg masses per plant, and the number of eggs per gram of root tissues. Statistically significant differences between overexpression/silenced plants and control plants expressing the empty vectors were determined using analysis of variance with *P* values less than 0.05 considered statistically significant.

### Subcellular and colocalization assays

For subcellular localization, the coding sequences of *Mi1D08B* ^*‐sp*^ and *NINJA‐family mc410* were first cloned into the Gateway entry vector pDONR221 (Thermo Fisher Scientific) using the BP Clonase. The entry clones were then used to generate C‐terminal fusions with eYFP in the destination vector pGWB441 using the LR Clonase. Primers used for cloning are listed in Table S2. The fusion constructs were confirmed by sequencing and introduced into *Agrobacterium tumefaciens* strain *EHA105*. *Agrobacterium* cultures were resuspended in infiltration buffer containing 10 mM MES, 10 mM MgCl_2_, and 0.15 mM acetosyringone, and infiltrated into leaves of six‐week‐old *Nicotiana benthamiana* plants with ER‐rb marker (CD3‐960) (Nelson et al., 2007). Approximately 72‐h post‐infiltration, YFP fluorescence was examined using a Leica SP8 confocal laser scanning microscope. For co‐localization assays, *Mi1D08B* ^*‐sp*^ and *NINJA‐family mc410* were cloned into pGWB441 and pGWB444 to generate eYFP and eCFP fusion constructs, respectively. *Agrobacterium* cultures harboring these constructs were adjusted to an OD_600_ of 1.0, mixed in equal proportions, and co‐infiltrated into *N. benthamiana* leaves. Fluorescence signals were observed at approximately 48‐h post‐infiltration using a Leica SP8 confocal microscope to detect both YFP and CFP signals. Fluorophore excitation and emission parameters were set as follows: YFP (514 nm excitation; 519–570 nm emission), CFP (458 nm excitation; 463–507 nm emission), and mCherry (587 nm excitation; 597–669 nm emission).

### Promoter activity assay

A fragment of 2341 bp upstream of the *mc410* start codon (ATG) was amplified from genomic DNA of the soybean cultivar Williams 82 by PCR and cloned upstream of the GUS reporter gene in a GFP‐containing vector pG2‐GUS as recently described (Zadegan et al., 2025). Primers used for cloning are listed in Table S2. The construct was confirmed by sequencing and transformed into *A. rhizogenes* strain *K599* to generate composite plants with transgenic hairy roots as described above. Transgenic roots were identified using GPF screening and then inoculated with approximately 200 J2 nematodes. Histochemical GUS staining was performed at 4‐, 7‐, and 14‐day post‐inoculation (dpi) in infected and non‐infected transgenic roots using at least 10 replicated plants.

### Hormone quantification

Transgenic hairy roots overexpressing *Mi1D08B* ^*‐sp*^ effector or carrying the empty vector (control) were identified using GFP reporter and pooled into four biological replicates. The tissues were flash‐frozen in liquid nitrogen and ground to a fine powder. Aliquots from each biological sample were retained for quantification of *Mi1D08B* expression. The remaining materials were sent to the Proteomics and Mass Spectrometry Facility at the Donald Danforth Plant Science Center (St. Louis, MO, USA) for hormone analysis. Hormone quantification was performed using an Eksigent ekspert™ microLC200 system coupled to a Sciex 6500 QTrap® mass spectrometer (Framingham, MA, USA).

### LC–MS/MS analysis

Fresh ground root tissues (100 mg) collected from *Mi1D08B*‐overexpressing and control soybean plants were extracted with 1 mL of 80% methanol, vortexed 30 s, and centrifuged at 15000**g** for 15 min at 4°C. Supernatants were collected for LC–MS/MS analysis. Aliquots (150 μL) were transferred to autosampler vials and submitted to the Biological Small Molecule Mass Spectrometry Core Facility, University of Tennessee, Knoxville (RRID: SCR 021368) for analysis. Metabolites were quantified using a method adapted from previously described Rabinowitz laboratory protocols with minor modifications (Chen et al., 2021; Rabinowitz & Kimball, 2007). Chromatography was performed on a Vanquish UHPLC with an Xbridge BEH Amide column (2.5 μm, 150 × 2 mm). Mobile phase A consisted of 95:5 H_2_O: acetonitrile with 20 mM ammonium acetate and 20 mM ammonium hydroxide (pH 9.4); mobile phase B was 100% acetonitrile. A volume of 1 μL was injected and subjected to the following gradient program (%B): 0–2 min 90; 3–7 min 75; 8–9 min 70; 10–12 min 50; 13–14 min 25; 16–20.5 min 0; 21–25 min 90; flow 0.150 mL min^−1^ with total runtime of 25 min. Mass spectrometry was performed on an Orbitrap Exploris™ 120 equipped with an ESI source operating in a positive mode (DDA). Full‐scan MS spectra were acquired over m/z 100–1000 at 120000 resolution (200 m/z), and MS^2^ spectra at 30000 resolution using stepped collision energies of 25/50/75 eV. Source parameters included spray voltage 3.5 kV, sheath gas 35 (arb), aux gas 7 (arb), ion transfer tube 320 °C. Raw files (.raw, Xcalibur) were converted to .mzML format using ProteoWizard MSConvert (Martens et al., 2011).

### Data processing and annotation

LC–MS/MS data were processed using MetaboAnalystR 4.0 and OptiLCMS (Pang et al., 2024). MS^1^ features were detected, aligned, and quantified, and MS^2^ data were used for deconvolution and HMDB, MoNA Series, LipidBlast, MassBank, GNPS, LipidBank, MINEs, LipidMAPS, and KEGG. Phytohormone‐related features (m/z, retention time, and intensity) were annotated using HormonomicsDB v1.4.1 (Giebelhaus et al., 2024) against the “PGR M + H” dataset in positive ionization mode with a 5 ppm mass tolerance. The resulting annotations were manually curated to distinguish metabolites that represent key intermediates and products of the OPDA‐to‐JA conversion pathway and remove analytical artifacts. When multiple mass features corresponded to the same compound identity, features broadly distributed across the chromatographic gradient and exhibiting low abundance were excluded as background ions. The feature with the lowest ppm error and the closest retention‐time match to the reference library method was selected as the best annotation candidate.

### Reverse transcription‐quantitative PCR (RT‐qPCR) analysis

Total RNA was extracted from approximately 300 mg of frozen, ground root tissues using TRIzol reagent (Invitrogen) and treated with DNase I (Invitrogen) to remove genomic DNA contamination. DNase‐treated RNA was used for gene expression analysis with the One‐Step RT‐PCR kit (Takara) following the manufacturer's instructions. Expression levels of *GmLOX6* (G*lyma.08G102900*), *GmAOS* (*Glyma.14 g078600*), *GmAOC4* (*Glyma.01 g086900*), *GmOPR2* (*Glyma.01 g235600*), *GmOPR3* (*Glyma.17 g050000*), *GmJAR4* (*Glyma.07 g057900*), *GmVSP‐B* (*Glyma.08G200100*), *GmAAT* (*Glyma.10G109500*), *GmPR1* (*Glyma.13 g252400*), and *GmPR2* (Glyma.07G104500) were quantified using three biological samples, each with three technical replicates. Both *β‐actin* (*Glyma.15G050200*) and soybean *ubiquitin* (*Glyma.20G141600*) were used as reference genes for normalization of expression levels (Hawk et al., 2024; Rambani et al., 2020). Primer sequences used for quantitative RT‐qPCR are listed in Table S2.

## Author Contributions

PL performed most of the experiments, analyzed data, and wrote the first draft. SBZ, NC, NG, AM, and MB contributed to nematode susceptibility assays, gene expression analysis, and in planta protein interaction assays. TB‐S contributed to hormone quantification and data analysis. VP contributed to project conceptualization and to manuscript editing. TH designed and supervised the experimental work, performed data analysis, and wrote the final version of the manuscript. All authors contributed to the study and approved the submitted version of the manuscript.

## Conflicts of Interest

The authors declare no conflicts of interest.

## Supporting information


**Figure S1.** Gene expression levels of *Mi1D08B* and *mc410* in overexpression and silenced plants. (A) RT‐qPCR quantification of transcript levels of *Mi1D08B* in transgenic hairy roots overexpressing *Mi1D08B* relative the endogenous reference gene actin, which was set to 1. (B, C) RT‐qPCR quantification of transcript levels of *mc410* in *mc410*‐overexpression (B) and *mc410*‐silenced plants (C). Relative fold‐change values represent gene expression levels in *mc410*‐overexpression or *mc410*‐silenced plants compared with the corresponding empty vector controls, which were set to 1. Asterisks indicate statistically significant differences from the empty vector controls as determined by *t*‐tests (*P* < 0.05). Bars represent mean ± SE from three independent biological replicates of root tissues, each analyzed with three technical replicates.

**Figure S2.** *Mi1D08B* and *mc410* modulate SA‐dependent defense gene expression in soybean roots. (A, B) RT‐qPCR quantification of transcript levels of the SA‐responsive marker genes *PR1* and *PR2* in *Mi1D08B*‐overexpressing (A) and *mc410*‐overexpressing roots (B) compared with their respective empty‐vector controls. Both genes are significantly downregulated in the overexpression lines. (C) RT‐qPCR quantification of transcript levels of *PR1* and *PR2* in *mc410*‐silenced roots (TRV2‐*mc410*), showing significant upregulation relative to TRV2 control roots. Bars represent mean ± SE from three independent biological replicates of root tissues, each analyzed with three technical replicates. Relative fold‐change values indicate gene expression levels in *Mi1D08B*‐overexpressing, *mc410*‐overexpressing, or *mc410*‐silenced plants compared with the corresponding empty vector controls, which were set to 1. Asterisks indicate statistically significant differences from the empty vector controls as determined by *t*‐tests (*P* < 0.05).

**Table S1.** Raw data for gall numbers, egg mass numbers, and eggs per gram of root in *Mi1D08B*‐overexpressing, *mc410*‐overexpressing, and *mc410*‐silenced roots.
**Table S2.** List of primer sequences used in this study.

## Data Availability

The data that support the findings of this study are included in the paper or its supplementary data.

## References

[tpj71095-bib-0001] Bali, S. & Gleason, C. (2024) Unveiling the diversity: plant parasitic nematode effectors and their plant interaction partners. Molecular Plant‐Microbe Interactions, 37(3), 179–189. Available from: 10.1094/MPMI-09-23-0124-FI 37870371

[tpj71095-bib-0002] Berliner, J. , Ganguly, A.K. , Kamra, A. , Sirohi, A. & Vp, D. (2023) Effect of elevated carbon dioxide on population growth of root‐knot nematode, *Meloidogyne incognita* in tomato. Indian Phytopathology, 76, 309–315. Available from: 10.1007/s42360-022-00584-8

[tpj71095-bib-0003] Bird, A.F. (1959) The attractiveness of roots to the plant parasitic nematodes *Meloidogyne javanica* and *M. hapla* . Nematologica, 4(4), 322–335. Available from: 10.1163/187529259X00534

[tpj71095-bib-0082] Boevink, P.C. , Wang, X. , McLellan, H. , He, Q. , Naqvi, S. , Armstrong, M.R. et al. (2016) A *Phytophthora infestans* RXLR effector targets plant PP1c isoforms that promote late blight disease. Nature Communications, 7, 10311. Available from: 10.1038/ncomms10311 PMC4740116 26822079

[tpj71095-bib-0004] Browse, J. (2009) Jasmonate passes muster: a receptor and targets for the defense hormone. Annual Review of Plant Biology, 60, 183–205. Available from: 10.1146/annurev.arplant.043008.092007 19025383

[tpj71095-bib-0005] Chen, C. , Hu, W. , Chen, C. , Qin, X. , Yu, J. , Jiang, Y. et al. (2025) A nematode effector, MiMSP8, targets U2AF, a subunit of a splicing factor involved in host pre‐mRNA splicing, thereby promoting parasitism. Plant, Cell & Environment, 48(9), 6847–6864. Available from: 10.1111/pce.15661 40458975

[tpj71095-bib-0006] Chen, J. , Lin, B. , Huang, Q. , Hu, L. , Zhuo, K. & Liao, J. (2017) A novel *Meloidogyne graminicola* effector, MgGPP, is secreted into host cells and undergoes glycosylation in concert with proteolysis to suppress plant defenses and promote parasitism. PLoS Pathogens, 13(4), e1006301. Available from: 10.1371/journal.ppat.1006301 28403192 PMC5402989

[tpj71095-bib-0007] Chen, L. , Lu, W. , Wang, L. , Xing, X. , Chen, Z. , Teng, X. et al. (2021) Metabolite discovery through global annotation of untargeted metabolomics data. Nature Methods, 18, 1377–1385. Available from: 10.1038/s41592-021-01303-3 34711973 PMC8733904

[tpj71095-bib-0008] Chini, A. , Gimenez‐Ibanez, S. , Goossens, A. & Solano, R. (2016) Redundancy and specificity in jasmonate signalling. Current Opinion in Plant Biology, 33, 147–156. Available from: 10.1016/j.pbi.2016.07.005 27490895

[tpj71095-bib-0009] Chini, A. , Monte, I. , Zamarreño, A.M. , Hamberg, M. , Lassueur, S. , Reymond, P. et al. (2018) An OPR3‐independent pathway uses 4, 5‐didehydrojasmonate for jasmonate synthesis. Nature Chemical Biology, 14(2), 171–178. Available from: 10.1038/nchembio.2540 29291349

[tpj71095-bib-0010] Crop Protection Network . (2024) Soybean disease loss calculator [online tool]. https://loss.cropprotectionnetwork.org/crops/soybean‐diseases

[tpj71095-bib-0011] Duplessis, S. , Cuomo, C.A. , Lin, Y.C. , Aerts, A. , Tisserant, E. , Veneault‐Fourrey, C. et al. (2011) Obligate biotrophy features unraveled by the genomic analysis of rust fungi. Proceedings of the National Academy of Sciences, 108(22), 9166–9171. Available from: 10.1073/pnas.1019315108 PMC3107277 21536894

[tpj71095-bib-0012] Escobar, C. , Barcala, M. , Cabrera, J. & Fenoll, C. (2015) Overview of root‐knot nematodes and giant cells. In: Advances in Botanical Research, Vol. 73. Academic Press, pp. 1–32. Available from: 10.1016/bs.abr.2015.01.001

[tpj71095-bib-0013] Feng, T. , Chen, Y. , Li, Z. , Pei, J. , Peng, D. , Peng, H. et al. (2024) A novel chorismate mutase effector secreted from root‐knot nematode *Meloidogyne enterolobii* manipulates plant immunity to promote parasitism. Journal of Integrative Agriculture, 23(12), 4107–4119. Available from: 10.1016/j.jia.2023.11.039

[tpj71095-bib-0014] Ghini, R. , Hamada, E. , Pedro Júnior, M.J. , Marengo, J.A. & Gonçalves, R.R.D.V. (2008) Risk analysis of climate change on coffee nematodes and leaf miner in Brazil. Pesquisa Agropecuária Brasileira, 43(2), 187–194. Available from: 10.1590/S0100-204X2008000200005

[tpj71095-bib-0015] Giebelhaus, R.T. , Erland, L.A. & Murch, S.J. (2024) HormonomicsDB: a novel workflow for the untargeted analysis of plant growth regulators and hormones. F1000Research, 11, 1191. Available from: 10.12688/f1000research.124194.2 39221023 PMC11364965

[tpj71095-bib-0016] Gleason, C. , Leelarasamee, N. , Meldau, D. & Feussner, I. (2016) OPDA has key role in regulating plant susceptibility to the root‐knot nematode *Meloidogyne hapla* in *Arabidopsis* . Frontiers in Plant Science, 7, 1565. Available from: 10.3389/fpls.2016.01565 27822219 PMC5075541

[tpj71095-bib-0017] Gleason, C.A. , Liu, Q.L. & Williamson, V.M. (2008) Silencing a candidate nematode effector gene corresponding to the tomato resistance gene Mi‐1 leads to acquisition of virulence. Molecular Plant‐Microbe Interactions, 21(5), 576–585. Available from: 10.1094/MPMI-21-5-0576 18393617

[tpj71095-bib-0018] Han, G.Z. (2017) Evolution of jasmonate biosynthesis and signaling mechanisms. Journal of Experimental Botany, 68(6), 1323–1331. Available from: 10.1093/jxb/erw470 28007954

[tpj71095-bib-0019] Hawk, T.E. , Li, P. , Abbas, H.M.K. , Sultana, M.S. , Piya, S. , Coffey, N. et al. (2026) A highly conserved SNARE‐associated protein enhances plant immunity by regulating vesicle trafficking. Plant Biotechnology Journal, 24, 3426–3440. Available from: 10.1111/pbi.70573 41636376 PMC13110161

[tpj71095-bib-0020] Hawk, T.E. , Piya, S. , Sultana, M.S. , Zadegan, S.B. , Shipp, S. , Coffey, N. et al. (2024) Soybean MKK2 establishes intricate signalling pathways to regulate soybean response to cyst nematode infection. Molecular Plant Pathology, 25(5), e13461. Available from: 10.1111/mpp.13461 38695657 PMC11064803

[tpj71095-bib-0021] Hewezi, T. (2015) Cellular signaling pathways and posttranslational modifications mediated by nematode effector proteins. Plant Physiology, 169(2), 1018–1026. Available from: 10.1104/pp.15.00923 26315856 PMC4587465

[tpj71095-bib-0022] Hewezi, T. (2024) Phytopathogens reprogram host alternative mRNA splicing. Annual Review of Phytopathology, 62(1), 173–192. Available from: 10.1146/annurev-phyto-121423-041908 38691872

[tpj71095-bib-0023] Hewezi, T. & Baum, T.J. (2013) Manipulation of plant cells by cyst and root‐knot nematode effectors. Molecular Plant‐Microbe Interactions, 26(1), 9–16. Available from: 10.1094/MPMI-05-12-0106-FI 22809272

[tpj71095-bib-0024] Hewezi, T. & Baum, T.J. (2017) Communication of sedentary plant‐parasitic nematodes with their host plants. Advances in Botanical Research, 82, 305–324. Available from: 10.1016/bs.abr.2016.11.004

[tpj71095-bib-0025] Hewezi, T. , Howe, P. , Maier, T.R. , Hussey, R.S. , Mitchum, M.G. , Davis, E.L. et al. (2008) Cellulose binding protein from the parasitic nematode *Heterodera schachtii* interacts with *Arabidopsis* pectin methylesterase: cooperative cell wall modification during parasitism. The Plant Cell, 20(11), 3080–3093. Available from: 10.1105/tpc.108.063065 19001564 PMC2613657

[tpj71095-bib-0026] Hewezi, T. , Juvale, P.S. , Piya, S. , Maier, T.R. , Rambani, A. , Rice, J.H. et al. (2015) The cyst nematode effector protein 10A07 targets and recruits host posttranslational machinery to mediate its nuclear trafficking and to promote parasitism in *Arabidopsis* . The Plant Cell, 27(3), 891–907. Available from: 10.1105/tpc.114.135327 25715285 PMC4558665

[tpj71095-bib-0027] Hu, Y. & Hewezi, T. (2018) Nematode‐secreted peptides and host factor mimicry. Journal of Experimental Botany, 69(12), 2866–2868. Available from: 10.1093/jxb/ery144 29846675 PMC5972653

[tpj71095-bib-0028] Huang, G. , Allen, R. , Davis, E.L. , Baum, T.J. & Hussey, R.S. (2006) Engineering broad root‐knot resistance in transgenic plants by RNAi silencing of a conserved and essential root‐knot nematode parasitism gene. Proceedings of the National Academy of Sciences, 103(39), 14302–14306. Available from: 10.1073/pnas.0604698103 PMC1570184 16985000

[tpj71095-bib-0029] Huang, G. , Dong, R. , Maier, T. , Allen, R. , Davis, E.L. , Baum, T.J. et al. (2004) Use of solid‐phase subtractive hybridization for the identification of parasitism gene candidates from the root‐knot nematode *Meloidogyne incognita* . Molecular Plant Pathology, 5(3), 217–222. Available from: 10.1111/j.1364-3703.2004.00220.x 20565611

[tpj71095-bib-0030] Jaouannet, M. , Magliano, M. , Arguel, M.J. , Gourgues, M. , Evangelisti, E. , Abad, P. et al. (2013) The root‐knot nematode calreticulin mi‐CRT is a key effector in plant defense suppression. Molecular Plant‐Microbe Interactions, 26(1), 97–105. Available from: 10.1094/MPMI-05-12-0130-R 22857385

[tpj71095-bib-0031] Johnson, L.Y. , Major, I.T. , Chen, Y. , Yang, C. , Vanegas‐Cano, L.J. & Howe, G.A. (2023) Diversification of JAZ‐MYC signaling function in immune metabolism. New Phytologist, 239(6), 2277–2291. Available from: 10.1111/nph.19114 37403524 PMC10528271

[tpj71095-bib-0032] Jones, J.T. , Haegeman, A. , Danchin, E.G. , Gaur, H.S. , Helder, J. , Jones, M.G. et al. (2013) Top 10 plant‐parasitic nematodes in molecular plant pathology. Molecular Plant Pathology, 14(9), 946–961. Available from: 10.1111/mpp.12057 23809086 PMC6638764

[tpj71095-bib-0033] Kantor, C. , Teixeira, M. , Kantor, M. & Gleason, C. (2025) Tiny invaders, big trouble: emerging nematode threats in the United States. Phytopathology, 115(6), 587–595. Available from: 10.1094/PHYTO-09-24-0290-IA 39961028

[tpj71095-bib-0034] Kim, J. , Yang, R. , Chang, C. , Park, Y. & Tucker, M.L. (2018) The root‐knot nematode *Meloidogyne incognita* produces a functional mimic of the *Arabidopsis* INFLORESCENCE DEFICIENT IN ABSCISSION signaling peptide. Journal of Experimental Botany, 69(12), 3009–3021. Available from: 10.1093/jxb/ery135 29648636 PMC5972575

[tpj71095-bib-0035] Kim, Y.H. , Park, C.W. , Lee, K.M. , Hong, C.O. , Son, H.J. , Kim, K.K. et al. (2025) The roles of MYC2 transcription factor in JA‐signaling pathway in plants. Journal of Plant Biology, 68(2), 113–131. Available from: 10.1007/s12374-025-09462-y

[tpj71095-bib-0036] Kunkel, B.N. & Brooks, D.M. (2002) Cross talk between signaling pathways in pathogen defense. Current Opinion in Plant Biology, 5(4), 325–331. Available from: 10.1016/S1369-5266(02)00275-3 12179966

[tpj71095-bib-0037] León, J. & Sánchez‐Serrano, J.J. (1999) Molecular biology of jasmonic acid biosynthesis in plants. Plant Physiology and Biochemistry, 37(5), 373–380. Available from: 10.1016/S0981-9428(99)80043-6

[tpj71095-bib-0038] Liu, J. , Zhang, J. , Wei, Y. , Su, W. , Li, W. , Wang, B. et al. (2024) The nematode effector calreticulin competes with the high mobility group protein OsHMGB1 for binding to the rice calmodulin‐like protein OsCML31 to enhance rice susceptibility to *Meloidogyne graminicola* . Plant, Cell & Environment, 47(5), 1732–1746. Available from: 10.1111/pce.14848 38311858

[tpj71095-bib-0039] Lozano‐Torres, J.L. , Wilbers, R.H. , Gawronski, P. , Boshoven, J.C. , Finkers‐Tomczak, A. , Cordewener, J.H. et al. (2012) Dual disease resistance mediated by the immune receptor Cf‐2 in tomato requires a common virulence target of a fungus and a nematode. Proceedings of the National Academy of Sciences, 109(25), 10119–10124. Available from: 10.1073/pnas.1202867109 PMC3382537 22675118

[tpj71095-bib-0040] Martens, L. , Chambers, M. , Sturm, M. , Kessner, D. , Levander, F. , Shofstahl, J. et al. (2011) mzML—a community standard for mass spectrometry data. Molecular & Cellular Proteomics, 10(1), R110.000133. Available from: 10.1074/mcp.R110.000133 PMC3013463 20716697

[tpj71095-bib-0041] Mejias, J. , Bazin, J. , Truong, N.M. , Chen, Y. , Marteu, N. , Bouteiller, N. et al. (2021) The root‐knot nematode effector MiEFF18 interacts with the plant core spliceosomal protein SmD1 required for giant cell formation. The New Phytologist, 229(6), 3408–3423. Available from: 10.1111/nph.17089 33206370

[tpj71095-bib-0042] Mejias, J. , Truong, N.M. , Abad, P. , Favery, B. & Quentin, M. (2019) Plant proteins and processes targeted by parasitic nematode effectors. Frontiers in Plant Science, 10, 970. Available from: 10.3389/fpls.2019.00970 31417587 PMC6682612

[tpj71095-bib-0043] Moens, M. , Perry, R.N. & Starr, J.L. (2009) *Meloidogyne* species‐a diverse group of novel and important plant parasites. In: Root‐Knot Nematodes. Wallingford, UK: CABI, pp. 1–17. Available from: 10.1079/9781845934927.0001

[tpj71095-bib-0083] Nelson, B.K. , Cai, X. & Nebenführ, A. (2007) A multicolored set of in vivo organelle markers for co‐localization studies in Arabidopsis and other plants. The Plant Journal, 51(6), 1126–1136. Available from: 10.1111/j.1365-313X.2007.03212.x 17666025

[tpj71095-bib-0044] Nguyen, C.N. , Perfus‐Barbeoch, L. , Quentin, M. , Zhao, J. , Magliano, M. , Marteu, N. et al. (2018) A root‐knot nematode small glycine and cysteine‐rich secreted effector, MiSGCR1, is involved in plant parasitism. New Phytologist, 217(2), 687–699. Available from: 10.1111/nph.14837 29034957

[tpj71095-bib-0045] Nilsson, A.K. , Fahlberg, P. , Johansson, O.N. , Hamberg, M. , Andersson, M.X. & Ellerström, M. (2016) The activity of HYDROPEROXIDE LYASE 1 regulates accumulation of galactolipids containing 12‐oxo‐phytodienoic acid in Arabidopsis. Journal of Experimental Botany, 67(17), 5133–5144. Available from: 10.1093/jxb/erw278 27422994 PMC5014160

[tpj71095-bib-0046] Niu, J. , Liu, P. , Liu, Q. , Chen, C. , Guo, Q. , Yin, J. et al. (2016) Msp40 effector of root‐knot nematode manipulates plant immunity to facilitate parasitism. Scientific Reports, 6(1), 19443. Available from: 10.1038/srep19443 26797310 PMC4726423

[tpj71095-bib-0047] Pang, Z. , Xu, L. , Viau, C. , Lu, Y. , Salavati, R. , Basu, N. et al. (2024) MetaboAnalystR 4.0: a unified LC‐MS workflow for global metabolomics. Nature. Communications, 15(1), 3675. Available from: 10.1038/s41467-024-48009-6 PMC11063062 38693118

[tpj71095-bib-0048] Pauwels, L. , Barbero, G.F. , Geerinck, J. , Tilleman, S. , Grunewald, W. , Pérez, A.C. et al. (2010) NINJA connects the co‐repressor TOPLESS to jasmonate signalling. Nature, 464(7289), 788–791. Available from: 10.1038/nature08854 20360743 PMC2849182

[tpj71095-bib-0049] Rabinowitz, J.D. & Kimball, E. (2007) Acidic acetonitrile for cellular metabolome extraction from *Escherichia coli* . Analytical Chemistry, 79(16), 6167–6173. Available from: 10.1021/ac070470c 17630720

[tpj71095-bib-0050] Rambani, A. , Hu, Y. , Piya, S. , Long, M. , Rice, J.H. , Pantalone, V. et al. (2020) Identification of differentially methylated miRNA genes during compatible and incompatible interactions between soybean and soybean cyst nematode. Molecular Plant‐Microbe Interactions, 33(11), 1340–1352. Available from: 10.1094/MPMI-07-20-0196-R 32757880

[tpj71095-bib-0051] Ratcliff, F. , Martin‐Hernandez, A.M. & Baulcombe, D.C. (2001) Technical advance: tobacco rattle virus as a vector for analysis of gene function by silencing. The Plant Journal, 25(2), 237–245. Available from: 10.1046/j.0960-7412.2000.00942.x 11169199

[tpj71095-bib-0052] Redkar, A. , Hoser, R. , Schilling, L. , Zechmann, B. , Krzymowska, M. , Walbot, V. et al. (2015) A secreted effector protein of *Ustilago maydis* guides maize leaf cells to form tumors. The Plant Cell, 27(4), 1332–1351. Available from: 10.1105/tpc.114.131086 25888589 PMC4558682

[tpj71095-bib-0054] Roychowdhury, R. , Hada, A. , Biswas, S. , Mishra, S. , Prusty, M.R. , Das, S.P. et al. (2025) Jasmonic acid (JA) in plant immune response: unravelling complex molecular mechanisms and networking of defence signalling against pathogens. Journal of Plant Growth Regulation, 44(1), 89–114. Available from: 10.1007/s00344-024-11264-4

[tpj71095-bib-0055] Rutter, W.B. , Franco, J. & Gleason, C. (2022) Rooting out the mechanisms of root‐knot nematode–plant interactions. Annual Review of Phytopathology, 60, 43–76. Available from: 10.1146/annurev-phyto-021621-120943 35316614

[tpj71095-bib-0056] Rutter, W.B. , Hewezi, T. , Maier, T.R. , Mitchum, M.G. , Davis, E.L. , Hussey, R.S. et al. (2014) Members of the *Meloidogyne* avirulence protein family contain multiple plant ligand‐like motifs. Phytopathology, 104(8), 879–885. Available from: 10.1094/PHYTO-11-13-0326-R 25014776

[tpj71095-bib-0057] Sato, M. , Tsuda, K. , Wang, L. , Coller, J. , Watanabe, Y. , Glazebrook, J. et al. (2010) Network modeling reveals prevalent negative regulatory relationships between signaling sectors in *Arabidopsis* immune signaling. PLoS Pathogens, 6(7), e1001011. Available from: 10.1371/journal.ppat.1001011 20661428 PMC2908620

[tpj71095-bib-0058] Saunders, D.G. , Win, J. , Cano, L.M. , Szabo, L.J. , Kamoun, S. & Raffaele, S. (2012) Using hierarchical clustering of secreted protein families to classify and rank candidate effectors of rust fungi. PLoS One, 7(1), e29847. Available from: 10.1371/journal.pone.0029847 22238666 PMC3253089

[tpj71095-bib-0059] Schornack, S. , van Damme, M. , Bozkurt, T.O. , Cano, L.M. , Smoker, M. , Thines, M. et al. (2010) Ancient class of translocated oomycete effectors targets the host nucleus. Proceedings of the National Academy of Sciences, 107(40), 17421–17426. Available from: 10.1073/pnas.1008491107 PMC2951462 20847293

[tpj71095-bib-0060] Sevier, C.S. & Kaiser, C.A. (2002) Formation and transfer of disulphide bonds in living cells. Nature Reviews Molecular Cell Biology, 3(11), 836–847. Available from: 10.1038/nrm954 12415301

[tpj71095-bib-0061] Sharma, H. & Chaubey, A.K. (2023) Plant parasitic nematodes: insights into the parasitic potential, adaptations and their interaction with other microorganisms. Nematodes‐Ecology, Adaptation and Parasitism. Available from: 10.5772/intechopen.1003258

[tpj71095-bib-0062] Shi, Q. , Liu, R. , Jiang, L. , Gao, S. , Ma, J. , Tian, X. et al. (2025) The nuclear effector MiISE23 from *Meloidogyne incognita* targets JAZ proteins and suppresses jasmonate signalling, increasing host susceptibility. Plant, Cell & Environment, 48(6), 4611–4624. Available from: 10.1111/pce.15461 40045540

[tpj71095-bib-0063] Shi, Q. , Mao, Z. , Zhang, X. , Ling, J. , Lin, R. , Zhang, X. et al. (2018) The novel secreted *Meloidogyne incognita* effector MiISE6 targets the host nucleus and facilitates parasitism in *Arabidopsis* . Frontiers in Plant Science, 9, 252. Available from: 10.3389/fpls.2018.00252 29628931 PMC5876317

[tpj71095-bib-0064] Shi, Q. , Mao, Z. , Zhang, X. , Zhang, X. , Wang, Y. , Ling, J. et al. (2018) A *Meloidogyne incognita* effector MiISE5 suppresses programmed cell death to promote parasitism in host plant. Scientific Reports, 8(1), 7256. Available from: 10.1038/s41598-018-24999-4 29740007 PMC5940819

[tpj71095-bib-0065] Song, H. , Lin, B. , Huang, Q. , Sun, T. , Wang, W. , Liao, J. et al. (2021) The *Meloidogyne javanica* effector Mj2G02 interferes with jasmonic acid signalling to suppress cell death and promote parasitism in *Arabidopsis* . Molecular Plant Pathology, 22(10), 1288–1301. Available from: 10.1111/mpp.13111 34339585 PMC8435226

[tpj71095-bib-0066] Soulé, S. , Huang, K. , Mulet, K. , Mejias, J. , Bazin, J. , Truong, N.M. et al. (2024) The root‐knot nematode effector MiEFF12 targets the host ER quality control system to suppress immune responses and allow parasitism. Molecular Plant Pathology, 25(7), e13491. Available from: 10.1111/mpp.13491 38961768 PMC11222708

[tpj71095-bib-0067] Sultana, M.S. , Niyikiza, D. , Hawk, T.E. , Coffey, N. , Lopes‐Caitar, V. , Pfotenhauer, A.C. et al. (2024) Differential transcriptome reprogramming induced by the soybean cyst nematode type 0 and type 1.2. 5.7 during resistant and susceptible interactions. Molecular Plant‐Microbe Interactions, 37(12), 828–840. Available from: 10.1094/MPMI-08-24-0092-R 39392447

[tpj71095-bib-0068] Thaler, J.S. , Humphrey, P.T. & Whiteman, N.K. (2012) Evolution of jasmonate and salicylate signal crosstalk. Trends in Plant Science, 17(5), 260–270. Available from: 10.1016/j.tplants.2012.02.010 22498450

[tpj71095-bib-0069] Truong, N.M. , Chen, Y. , Mejias, J. , Soulé, S. , Mulet, K. , Jaouannet, M. et al. (2021) The *Meloidogyne incognita* nuclear effector MiEFF1 interacts with *Arabidopsis* cytosolic glyceraldehyde‐3‐phosphate dehydrogenases to promote parasitism. Frontiers in Plant Science, 12, 641480. Available from: 10.3389/fpls.2021.641480 33897729 PMC8062903

[tpj71095-bib-0070] Tsai, A.Y.L. , Iwamoto, Y. , Tsumuraya, Y. , Oota, M. , Konishi, T. , Ito, S. et al. (2021) Root‐knot nematode chemotaxis is positively regulated by l‐galactose sidechains of mucilage carbohydrate rhamnogalacturonan‐I. Science Advances, 7(27), eabh4182. Available from: 10.1126/sciadv.abh4182 34215589 PMC11060035

[tpj71095-bib-0071] Verhoeven, A. , Finkers‐Tomczak, A. , Prins, P. , Valkenburg‐van Raaij, D.R. , Van Schaik, C.C. , Overmars, H. et al. (2023) The root‐knot nematode effector MiMSP32 targets host 12‐oxophytodienoate reductase 2 to regulate plant susceptibility. New Phytologist, 237(6), 2360–2374. Available from: 10.1111/nph.18653 36457296

[tpj71095-bib-0072] Wang, D. , Tian, L. , Zhang, D.D. , Song, J. , Song, S.S. , Yin, C.M. et al. (2020) Functional analyses of small secreted cysteine‐rich proteins identified candidate effectors in *Verticillium dahliae* . Molecular Plant Pathology, 21(5), 667–685. Available from: 10.1111/mpp.12921 32314529 PMC7170778

[tpj71095-bib-0073] Wieczorek, K. (2015) Cell wall alterations in nematode‐infected roots. In: Advances in Botanical Research, Vol. 73. Academic Press, pp. 61–90. Available from: 10.1016/bs.abr.2014.12.002

[tpj71095-bib-0074] Yi, R. , Li, Y. & Shan, X. (2024) OPDA/dn‐OPDA actions: biosynthesis, metabolism, and signaling. Plant Cell Reports, 43(8), 206. Available from: 10.1007/s00299-024-03286-9 39093416

[tpj71095-bib-0075] Yu, J. , Yuan, Q. , Chen, C. , Xu, T. , Jiang, Y. , Hu, W. et al. (2024) A root‐knot nematode effector targets the *Arabidopsis* cysteine protease RD21A for degradation to suppress plant defense and promote parasitism. The Plant Journal, 118(5), 1500–1515. Available from: 10.1111/tpj.16692 38516730

[tpj71095-bib-0076] Zadegan, S.B. , Abbas, H.M.K. , Piya, S. , Hawk, T.E. , Lopes‐Caitar, V. , Adams, N. et al. (2026) An avirulence‐associated small secreted effector from *Meloidogyne incognita* enhances tomato resistance via lipoxygenase targeting. Journal of Experimental Botany, 77, erag049. Available from: 10.1093/jxb/erag049 41612949

[tpj71095-bib-0077] Zadegan, S.B. , Li, P. , Sultana, M.S. , Abbas, H.M.K. , Coffey, N. , Öztürk, C. et al. (2025) Single‐nucleus RNA‐sequencing reveals the cellular programs driving nematode‐induced giant cell formation in tomato. Horticulture Research, 12(11), uhaf223. Available from: 10.1093/hr/uhaf223 41209832 PMC12596086

[tpj71095-bib-0078] Zhang, X. , Nguyen, N. , Breen, S. , Outram, M.A. , Dodds, P.N. , Kobe, B. et al. (2017) Production of small cysteine‐rich effector proteins in *Escherichia coli* for structural and functional studies. Molecular Plant Pathology, 18(1), 141–151. Available from: 10.1111/mpp.12385 26915457 PMC6638209

[tpj71095-bib-0079] Zhao, J. , Huang, K. , Liu, R. , Lai, Y. , Abad, P. , Favery, B. et al. (2024) The root‐knot nematode effector Mi2G02 hijacks a host plant trihelix transcription factor to promote nematode parasitism. Plant Communications, 5(2), 100723. Available from: 10.1016/j.xplc.2023.100723 37742073 PMC10873892

[tpj71095-bib-0080] Zhao, J. , Mejias, J. , Quentin, M. , Chen, Y. , de Almeida‐Engler, J. , Mao, Z. et al. (2020) The root‐knot nematode effector MiPDI1 targets a stress‐associated protein (SAP) to establish disease in Solanaceae and *Arabidopsis* . New Phytologist, 228(4), 1417–1430. Available from: 10.1111/nph.16745 32542658

[tpj71095-bib-0081] Zhou, X.E. , Zhang, Y. , Yao, J. , Zheng, J. , Zhou, Y. , He, Q. et al. (2023) Assembly of JAZ–JAZ and JAZ–NINJA complexes in jasmonate signaling. Plant Communications, 4(6), 100639. Available from: 10.1016/j.xplc.2023.100639 37322867 PMC10721472

