# Supplementary material for: The root‐knot nematode effector Mi1D08B targets a NINJA‐family co‐repressor to suppress jasmonate production and promote infection in soybean
Source: Plant J. 2026 Aug 22;127(4):e71095. doi: 10.1111/tpj.71095 (PMC13499025; doi:10.1111/tpj.71095)
Supplement: Supplementary file 1 [file TPJ-127-0-s001.pdf]

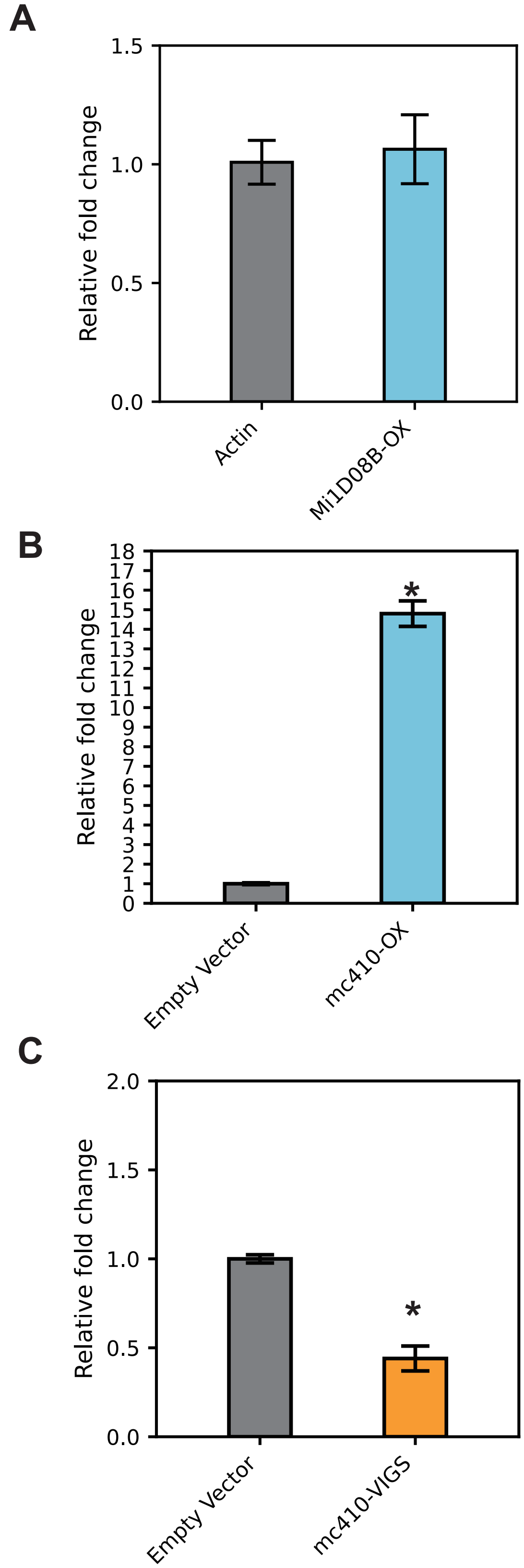

Supplemental Figure 1. Gene expression levels of *Mi1D08B* and *mc410* in overexpression and silenced plants.

A: RT-qPCR quantification of transcript levels of *Mi1D08B* in transgenic hairy roots overexpressing *Mi1D08B* relative the endogenous reference gene actin, which was set to 1.

B and C: RT-qPCR quantification of transcript levels of *mc410* in *mc410*-overexpression (B) and *mc410*-silenced plants (C). Relative fold-change values represent gene expression levels in *mc410*-overexpression or *mc410*-silenced plants compared with the corresponding empty vector controls, which were set to 1. Asterisks indicate statistically significant differences from the empty vector controls as determined by t-tests ($P < 0.05$). Bars represent mean ± SE from three independent biological replicates of root tissues, each analyzed with three technical replicates.
