# Supplementary material for: The root‐knot nematode effector Mi1D08B targets a NINJA‐family co‐repressor to suppress jasmonate production and promote infection in soybean
Source: Plant J. 2026 Aug 22;127(4):e71095. doi: 10.1111/tpj.71095 (PMC13499025; doi:10.1111/tpj.71095)
Supplement: Supplementary file 2 [file TPJ-127-0-s003.pdf]

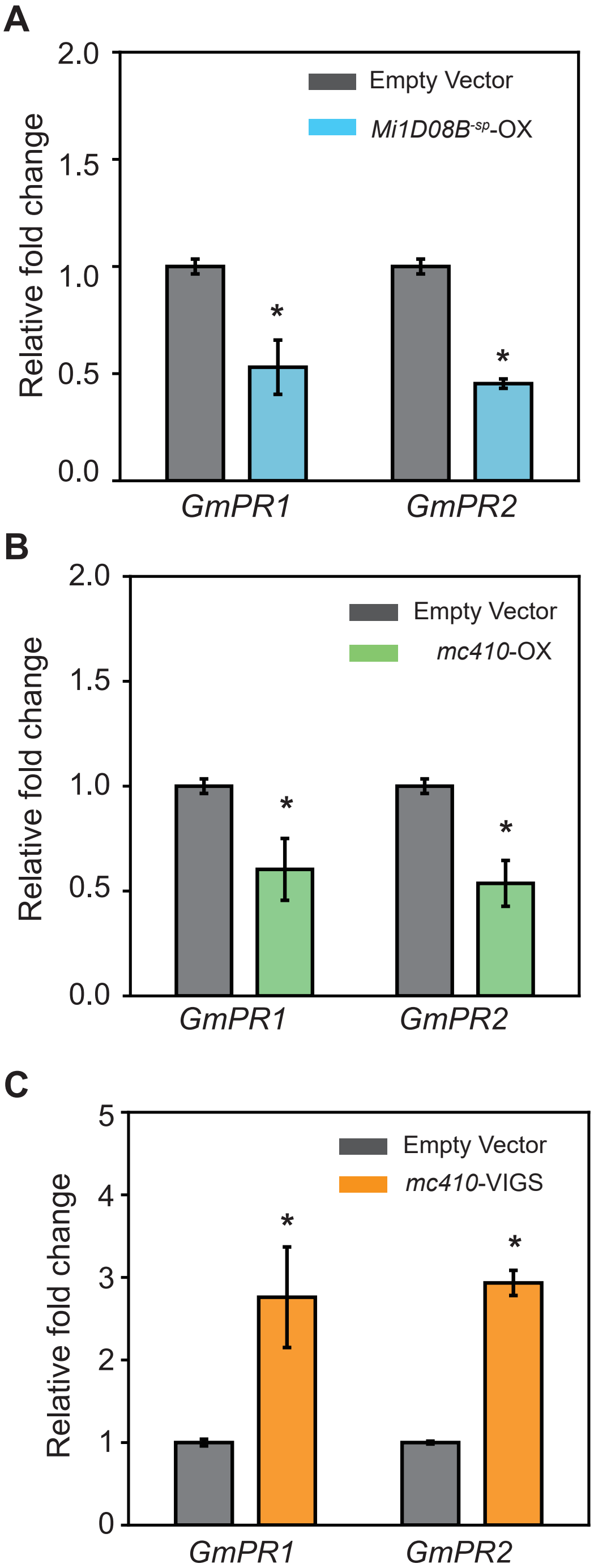

Supplemental Figure 2. Mi1D08B and mc410 modulate SA-dependent defense gene expression in soybean roots.

A and B: RT-qPCR quantification of transcript levels of the SA-responsive marker genes *PR1* and *PR2* in *Mi1D08B*-overexpressing (A) and *mc410*-overexpressing roots (B) compared with their respective empty-vector controls. Both genes are significantly downregulated in the overexpression lines.
C. RT-qPCR quantification of transcript levels of *PR1* and *PR2* in *mc410*-silenced roots (TRV2-*mc410*), showing significant upregulation relative to TRV2 control roots.
Bars represent mean ± SE from three independent biological replicates of root tissues, each analyzed with three technical replicates. Relative fold-change values indicate gene expression levels in *Mi1D08B*-overexpressing, *mc410*-overexpressing, or *mc410*-silenced plants compared with the corresponding empty vector controls, which were set to 1. Asterisks indicate statistically significant differences from the empty vector controls as determined by t-tests ($P < 0.05$).
